# Molecular Basis of Bicyclic Boronate β-Lactamase Inhibitors of Ultrabroad Efficacy – Insights From Molecular Dynamics Simulation Studies

**DOI:** 10.3389/fmicb.2021.721826

**Published:** 2021-08-04

**Authors:** Emilio Lence, Concepción González-Bello

**Affiliations:** Departamento de Química Orgánica, Centro Singular de Investigación en Química Biolóxica e Materiais Moleculares, Universidade de Santiago de Compostela, Santiago de Compostela, Spain

**Keywords:** boron-based β-lactamase inhibitors, simulations of biomacromolecules, metallo-β-lactamases, serine-β-lactamases, binding mode, QPX7728, taniborbactam, VNRX-5133

## Abstract

β-Lactam antibiotics represent about 70% of all antibacterial agents in clinical use. They are safe and highly effective drugs that have been used for more than 50 years, and, in general, well tolerated by most patients. However, its usefulness has been dramatically reduced with the spread and dissemination worldwide of multi-drug resistant bacteria. These pathogens elude the therapeutic action of these antibiotics by expressing β-lactamase enzymes that catalyze the hydrolysis of their β-lactam ring to give inactive products, which is one of the most relevant resistance mechanisms in deadly pathogens such as *Pseudomonas aeruginosa*, *Acinetobacter baumannii*, and *Enterobacteriaceae*. From the drug development point of view, the design of an efficient β-lactamase inhibitor able to block this antibiotic resistance mechanism and restore β-lactam antibiotics efficacy is challenging. This is due to: (1) the huge structural diversity of these enzymes in both the amino acid sequence and architecture of the active site; (2) the distinct hydrolytic capability against different types of substrates; (3) the variety of enzyme mechanisms of action employed, either involving covalent catalyzed processes (serine hydrolases) or non-covalent catalysis (zinc-dependent hydrolases); and (4) the increasing emergence and spread of bacterial pathogens capable of simultaneously producing diverse β-lactamases. Hence, a long-pursued goal has been the development of ultrabroad-spectrum inhibitors able to inhibit both serine- and metallo-β-lactamases. The recent development of taniborbactam (formerly VNRX-5133) and QPX7728, which are bicyclic boronate inhibitors currently under clinical development, represents a huge step forward in this goal. In this article, the molecular basis of the ultrabroad-spectrum of activity of these boron-based inhibitors is analyzed by molecular dynamics simulation studies using the available crystal structures in complex with both inhibitors, or the models constructed from wild-type forms. The efficacy of taniborbactam and QPX7728 is compared with the cyclic boronate inhibitor vaborbactam, which is the first boron-based β-lactamase inhibitor approved by the FDA in combination with meropenem for the treatment of complicated urinary tract infections.

## Introduction

The clinical utility of β-lactam antibiotics (penicillins, cephalosporins, monobactams, and carbapenems), which represent about 70% of all antibacterial drugs, is currently threatened by the worldwide proliferation and dissemination of bacteria producing β-lactamases. These enzymes hydrolyze their β-lactam ring to produce products that are inactive against their therapeutic target (Baren and Bradford, 2019, 2020). This deactivation mechanism represents the most prevalent cause of bacterial resistance to antibiotics in Gram-negative bacteria, in particular in the antibiotic-resistant “priority pathogens” identified by the World Health Organization (WHO) *Pseudomonas aeruginosa*, *Acinetobacter baumannii*, and *Enterobacteriaceae* [World Health Organization (WHO), 2017]. These pathogens are resistant to carbapenems, which are often considered as antibiotic of last resort, and are linked with the most dangerous healthcare-associated infections, specially on patients with a compromised immune system. To reduce the impact of β-lactamase producing bacteria and restore β-lactam antibiotics efficacy, the use of a β-lactamase inhibitor in combination with the antibiotic has proven to be very successful strategy (Tyers and Wright, 2019). Good examples are the widely used combination therapies, amoxycillin/clavulanic acid (approved 1984), ampicillin/sulbactam (approved 1986), and piperacillin/tazobactam (approved 1993). However, the increasing emergence and spread worldwide of evolved pathogens harboring more challenging and sophisticated new β-lactamases enzymes for which these combinations failed, has triggered an intense search in the last years for alternative inhibitors and combinations, which mainly has been carried out by biotechnology companies (Wright, 2016; Melander and Melander, 2017; Douafer et al., 2019; Hernando-Amado et al., 2019; González-Bello et al., 2020).

The challenges in the design and development of new inhibitors of β-lactamase enzymes is their enormous diversity and continuous evolution. Thus, until today, more than 7000 β-lactamases have been identified that are classified based on their amino acid sequence in four major classes, A–D (Naas et al., 2017). They are quite distinct enzymes, because of: (1) the varied architecture of the active site; (2) the distinct hydrolytic capability against different types of substrates; and (3) the mechanism of action employed. Thus, classes A, C, and D are serine-β-lactamase enzymes that hydrolyze the β-lactam ring of the antibiotic in a two-step covalent catalyzed process, involving the formation of an acyl-enzyme adduct through the nucleophilic attack of a catalytic serine residue, followed by a deacylation process by reaction with a water molecule. On the contrary, class B enzymes (metallo-β-lactamases) employ a non-covalent process using a Zn(II)-bound hydroxide anion as a nucleophile that attacks the β-lactam carbonyl group to give a tetrahedral intermediate, thus triggering the cleavage of the C–N bond to afford an anionic intermediate. The subsequent protonation of this intermediate provokes the release of the product for turnover. Class B enzymes are divided into three subclasses, B1, B2, and B3. Besides none of them hydrolyze monobactams, their hydrolytic capabilities and active site architecture are quite different. Thus, the B1 and B3 subclasses can hydrolyze virtually all β-lactam antibiotics and most of them have two catalytic Zn(II) ions. On the contrary, B2 enzymes are mono-zinc-dependent hydrolases and specifically hydrolyze carbapenems.

Among all β-lactamases, much effort is currently being devoted to the development of inhibitors against carbapenemases, since for those cases few or even no treatments are available (Papp-Wallace, 2019). These include: (1) all metallo-β-lactamases, such as IMP (B1), VIM (B1), NDM (B1), CphA (B2), and L1 (B3); and (2) serine-carbapenemases of classes A like KPC, SME, IMI, NMC-A, and GES-2, and of classes D such as and OXA-23, OXA-24/40, and OXA-48. So far, great success has been achieved in the inhibition of serine-carbapenemases of classes A and some class D with 1,6-diazabicyclo[3,2,1]octane (DBO) derivatives such as: (1) avibactam, which was approved in 2014 in combination with ceftazidime for the treatment of complicated intra-abdominal infections and in 2019 for hospital-acquired bacterial pneumonia and ventilator-associated bacterial pneumonia in patients 18 years and older. It is also under phase III clinical trials in combination with aztreonam (Papp-Wallace and Bonomo, 2016); and (2) durlobactam (formerly ETX2514) (Durand-Réville et al., 2017), which in combination with sulbactam is currently under phase III clinical studies.^1^ However, the development of metallo-β-lactamase inhibitors has been less successful, and currently it is an unmet medical need as no therapies are available. The biggest obstacles in this goal has been: (1) the marked differences in the mechanism of action between both types of β-lactamases that hinders the application of the extensive knowledge acquired in the inhibition of the serine-β-lactamases, such as with DBO inhibitors, to metallo-β-lactamases; (2) the architecture of the active site involving the presence of one or two Zn(II) ions with a very defined coordination pattern; and (3) the potential off-target effects of some of the earlier identified inhibitors [boronic acid derivatives (Cendron et al., 2019), 2,6-dipicolinic acid analogs (Chen et al., 2017), etc.] due to inhibition of mammalian serine proteases (Roll et al., 2010). In addition, the increasing emergence and worldwide spread of bacterial pathogens coproducing metallo- and serine-β-lactamases brings to light the urgent need for the development of β-lactamase inhibitors with ultrabroad-spectrum of activity capable to deal with any kind of carbapenemase produced by the pathogen, or at least efficient enough for most of them to restore antibiotic efficacy under different circumstances (Avolio et al., 2017; Contreras et al., 2020; Naha et al., 2021). The discovery of bicyclic boronate inhibitors represents a huge step forward in this long-pursued goal, not only because the effective inhibition of metallo-β-lactamases has been achieved with them, but also for the recently discovered inhibitors able to inhibit efficiently the carbapenem hydrolyzing class D β-lactamases present in *A. baumannii* (OXA-23, OXA-24/40).

**FIGURE 1:**
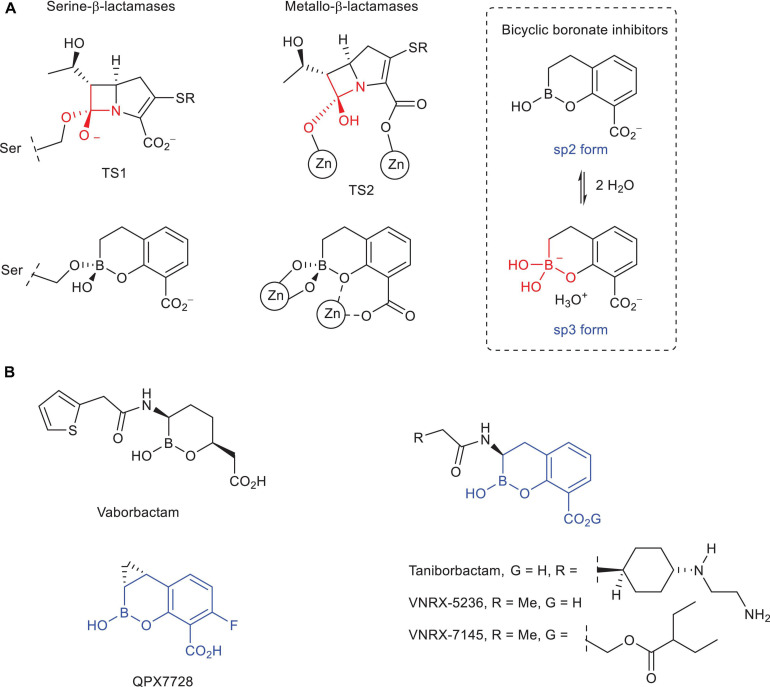
**(A)** Schematic representation of the transition state analogs in the carbapenem hydrolysis catalyzed by serine- and metallo-β-lactamase enzymes and the resulting bicyclic boronate adduct and di-Zn(II) enzyme complexes. The sp2/sp3 equilibrium forms of the bicyclic boronate inhibitors are also shown. **(B)** Chemical structures of vaborbactam (formerly RPX7009), taniborbactam (formerly VNRX-5133), VNRX-5236, VNRX-7145 (oral proform of VNRX-5236), and QPX7728.

Bicyclic boronate derivatives have proven to be good mimetics of both transition state analogs in serine- and metallo-β-lactamase catalyzed process, thanks to: (1) as for other boron-based analogs, their ability to change their hybridization state between sp2 (trigonal) and sp3 (tetrahedral) forms; and (2) its large conformational restraint (Figure 1). The latter feature is a key difference with vaborbactam, which is the first boron-based β-lactamase inhibitor approved by the FDA (2017) in combination with meropenem for the treatment of complicated urinary tract infections, but a weak inhibitor for both class B and carbapenem hydrolyzing class D β-lactamases (Tsivkovski and Lomovskaya, 2020). The most relevant bicyclic boronate inhibitors are taniborbactam (formerly VNRX-5133) (Krajnc et al., 2019; Liu et al., 2020) and QPX7728 (Hecker et al., 2020; Nelson et al., 2020; Sabet et al., 2020), which are currently under clinical development in combination with cefepime^2^ and QPX2014^3^ (chemical structure not yet disclosed), respectively. Both compounds inhibit most class B enzymes in the nanomolar range. The difference between them lies in the superior inhibitory capacity of QPX7728 against carbapenem hydrolyzing class D β-lactamases usually found in *A. baumannii* (OXA-23, OXA-24/40), which makes it an excellent pan-β-lactamase inhibitor. Since there is not full structural information about how these bicyclic boronate inhibitors modulate the enzymatic activity of these challenging enzymes, in this article, the molecular basis of the ultrabroad-spectrum of activity of QPX7728 is explored by molecular dynamics (MD) simulation studies. To this end, the formation of the enzyme-boronate adducts/complexes using models constructed from wild-type forms or in complex with other chemically different compounds (OXA-23, OXA-24/40), and the induced-fit plasticity of the available crystal structures in complex with these inhibitors (OXA-48) were explored. The efficacy of QPX7728 is compared with taniborbactam, VNRX-5236, which is the active form of the orally bioavailable prodrug VNRX-7145 (Burns et al., 2019; Hamrick et al., 2019) under clinical development,^4^ and vaborbactam. The hybridization state form required for inhibition is analyzed. In addition, the molecular basis of the experimentally observed selectivity of QPX7728 against IMP-1 metallo-carbapenemases is also explored.

## Results and Discussion

The inhibitory properties of QPX7728, taniborbactam and vaborbactam against carbapenem hydrolyzing class D β-lactamases usually found in *A. baumannii* (OXA-23, OXA-48) and class B enzymes are summarized on Table 1. These data show that: (1) among the three compounds, only QPX7728 shows sufficient inhibitory capacity against OXA-23 and OXA-48 enzymes for medical applications; (2) QPX7728 and taniborbactam are excellent inhibitors of VIM and NDM enzymes (subclass B1) but only QPX7728 inhibits sufficiently IMP enzymes; and (3) vaborbactam is insufficiently active against all these types of enzymes. From the structural point of view, the main differences between QPX7728 and taniborbactam are: (1) the greater rigidity of the bicyclic boronate group in QPX7728 due to the presence of an additional constrained three-membered ring (cyclopropane) on the position adjacent to the boron atom; (2) the lack of a flexible and long substituent in the aforementioned position, which is also located on the opposite face of the bicyclic boronate core; and (3) the introduction of an electron-withdrawing (fluoride) in the *ortho* position to the carboxylate group that reduces the electron density of the aromatic ring and/or promotes extra binding interactions with the nearby residues of the active site. It is important to note that the replacement of the fluoride by a methoxy group (donor) also restores efficiently the activity of the β-lactam antibiotic (meropenem, biapenem) *in vitro*, but this ability decreases in the absence of substituent (hydrogen) (Hecker et al., 2020). In addition, the pharmacological properties and metabolic liability of this type of bicyclic boronate inhibitors is enhanced with the substitution of the aforementioned position.

**TABLE 1:** Inhibitory activity [IC_50_ (μM), K_i_ (μM)] of QPX7728, taniborbactam, and vaborbactam against relevant carbapenemases.

		QPX7728		Taniborbactam		Vaborbactam	
Enzyme	Class	IC_50_ (μM)	K_i_ (μM)	IC_50_ (μM)	K_i_ (μM)	IC_50_ (μM)	K_i_ (μM)
OXA-48	D	0.0011^c^	0.00028^a^	2.39^b^	0.35^d^	32^e^	14^a^
OXA-23	D	0.0012^c^	0.00074^a^	NA	NA	120^c^	>40^a^
VIM-1	B1	0.014^c^	0.0080^a^	0.0079^b^	NA	398^e^	>40^a^
VIM-2	B1	NA	NA	0.0005^b^	0.019^d^	316^e^	NA
NDM-1	B1	0.055^c^	0.032^a^	0.01^b^	0.081^d^	631^e^	>40^a^
IMP-1	B1	0.610^c^	0.22^a^	2.51^b^	>30^d^	126^e^	>40^a^
CphA	B2	NA	NA	2.51^b^	NA	631^e^	NA
L1	B2	NA	NA	>10^b^	NA	336^e^	NA

*^*a*^Data from Hecker et al. (2020). ^*b*^Data from Krajnc et al. (2019). ^*c*^Data from Tsivkovski et al. (2020). ^*d*^Data from Hamrick et al. (2020). ^*e*^Data from Langley et al. (2019). NA, not available.*

### Interaction of QPX7728, Taniborbactam, VNRX-5236, and Vaborbactam Against Class D Carbapenemases (OXA-23, OXA-24/40, OXA-48)

For the class D carbapenamase enzymes, the crystal structure of *K. pneumoniae* OXA-48 inhibited by QPX7728 has been described (PDB ID 6V1O) (Hecker et al., 2020). However, no 3D structural information on the corresponding adduct with OXA-23 and OXA-24/40, as well as on the interaction of taniborbactam with these three enzymes are available. Therefore, to get an insight of the molecular basis of the experimentally observed differences in the inhibitory capacity of these bicyclic boronate inhibitors, we have taken the advantage of computational methods aimed at the simulation of biomacromolecules. Unlike computationally less demanding and more widely used docking studies (lock-and-key model), MD simulations considers the intrinsic-shape motion and conformational changes of the target induced by the ligand (induced-fit model), which is immersed in a water molecules box to also mimic the biological environment. This computational technique has proven to be an excellent tool for: (1) knowing in atomic detail the conformational changes induced by the ligand upon binding; (2) determining the key interactions responsible for the binding of a set of structurally diverse ligands, as well as the strength of those contacts; (3) exploring transitory complexes that otherwise would require the resolution of 3D crystal structures in multiple states, which are difficult to achieve in most cases; and (4) analyzing the binding mode of a variety of inhibitors and enzymes when experimental structural information cannot be obtained by X-ray crystallography, usually because no crystals with the suitable diffracting properties are available.

#### OXA-48 Enzyme

First, the inactivation of OXA-48 by taniborbactam, VNRX-5236, and vaborbactam was studied by docking and MD simulation studies, and the results were compared with the available crystal structure of OXA-48 modified by QPX7728 (PDB ID 6V1O). The difficulty of performing MD simulations on such molecules containing boron atoms is: (1) the lack of the force-field parameters required (non-bond, bond, angle, and dihedral); (2) the huge time effort to do full force-field parameterization manually; as well as (3) the few available protocols to perform this task by available programs. Perhaps this explains why there are not many examples of simulation studies involving ligands of this type. Herein, we have applied Seminario method (Seminario, 1996) implemented in the Visual Force Field Derivation Toolkit (VFFDT) (Zheng et al., 2016) and Paramfit programs recently reported (Kurt and Temel, 2020). This approach was first validated by studying the QPX7728@OXA-48 enzyme complex for inactivation (Michaelis complex) and the subsequent QPX7728@OXA-48 enzyme adduct, and the latter was compared with the experimentally obtained (PDB ID 6V1O).

**FIGURE 2:**
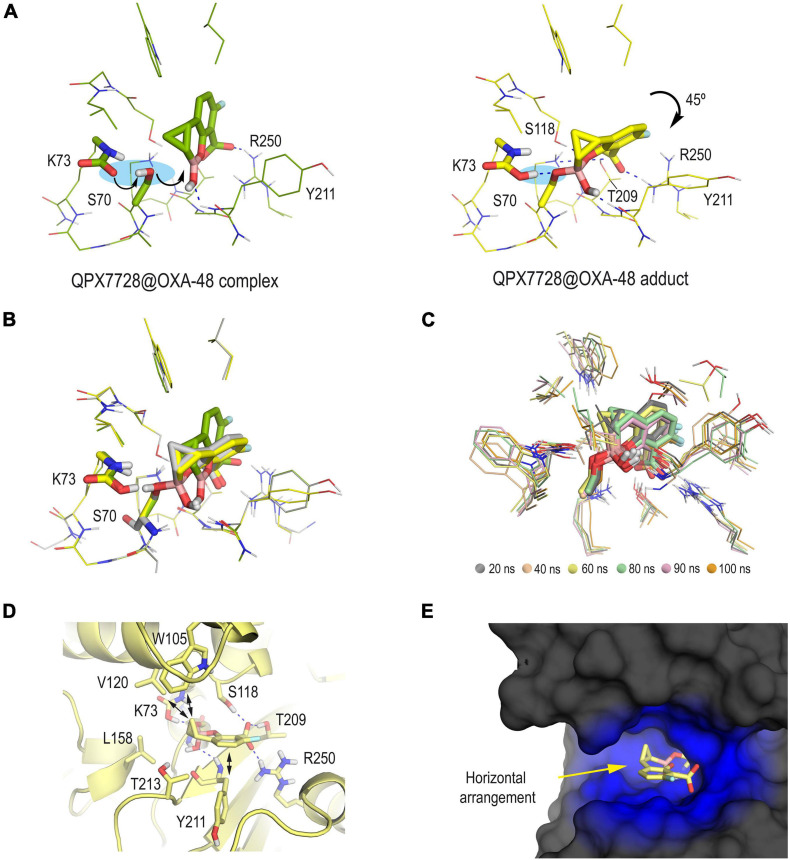
**(A)** QPX7728@OXA-48 complex (green) and QPX7728@OXA-48 adduct (yellow) obtained by computational studies. Note how the formation of the enzyme adduct would involve the deprotonation of the catalytic serine by the carboxylated residue K73 followed by nucleophilic attack to the boronate group. The bicycle moiety is inclined ∼45° after reaction. **(B)** Comparison of **(A)** and PDB ID 6V1O (gray). Note how the QPX7728@OXA-48 adduct (yellow) obtained by computational studies adequately reproduces the arrangement observed in the crystal structure, and therefore the carboxylated residue K73 would be in its protonated form. **(C)** Superposition of several snapshots from the 100 ns of MD simulation on the QPX7728@OXA-48 adduct. **(D,E)** Interactions and overall view of the modified QPX7728 in the active site. Note how the horizontal disposition of the modified QPX7728 facilitates CH-π and apolar interactions through both faces of the bicyclic moiety. Relevant side chain residues are shown and labeled. Key hydrogen bonding and electrostatic interactions are shown as blue dashed lines.

In addition to determining the protonation state of the ionizable residues, an important point here was to know which form of the bicyclic boronate scaffold, i.e., sp2 or sp3 forms, would trigger the formation of the serine adduct. To this end, the most suitable arrangements for both hybridization forms of QPX7728 in the active site were first explored by molecular docking. The program GOLD version 2020.2.0 (Jones et al., 1997) and the enzyme coordinates obtained in PDB ID 6V1O (chain A) were employed. The proposed QPX7728@OXA-48 enzyme complexes obtained by docking in a truncated octahedron of water molecules obtained with the molecular mechanics force field AMBER (Case et al., 2015) were then subjected to 100 ns of dynamic simulation. The outcomes of the studies performed using the sp3 form of QPX7728 showed an inadequate arrangement of both the catalytic serine residue S70 for nucleophilic attack and the ligand, which was located too far for nucleophilic addition (Supplementary Figure 1). This fact would be mainly due to the negative charge of the boron moiety since a 180° rotation of the ligand toward the positively charged residue R250 was observed during the simulation. On the contrary, the sp2 from of QPX7728 resulted to be located close to the catalytic serine and well anchored in the active site through strong hydrogen bonding interactions with the guanidinium group of R250, the side chain of T209 and the main NH group of Y211 (Figure 2A). More importantly, during the minimization step (prior simulation), the spontaneous deprotonation of the catalytic serine S70 by the carboxylated lysine residue K73, which is characteristic of this type of enzymes, was observed. The latter triggered the subsequent nucleophilic attack of S70 to the boronate moiety (sp2 form), leading to the QPX7728@OXA-48 enzyme adduct formation. The resulting covalently modified serine residue would also be stabilized by the protonated carboxylated residue K73 through hydrogen bonding interaction. Upon forming of the serine adduct, the ligand rotated ∼45° to rest on top of residue Y211, which is located on the outermost part of the active site (Figure 2B). The resulting arrangement agrees with that observed in the crystal structure PDB ID 6V1O and proved to be very stable as no significant changes in its position were observed during the whole simulation (100 ns) (Figure 2C). The horizontal disposition of the modified QPX7728 would facilitate CH-π and apolar interactions through both faces of the bicyclic moiety, specifically with residues W105 and V120 located on the active site-covering loop and with carbon side chain of residue Y211 (Figure 2D). As a result, the modified QPX7728 would be buried and surrounded by the enzyme (Figure 2E). Overall, these results suggested that: (1) the formation of the QPX7728@OXA-48 enzyme adduct would involve the sp2 form of the inhibitor and the reaction is expected to be very fast and with a low energy barrier; (2) the carboxylated residue K73 would trigger the formation of the serine adduct and would also contribute to the stabilization of the boronate enzyme adduct; and (3) the reaction with the catalytic serine promotes a 45° turn on the modified ligand to maximize its interactions from both faces of the bicyclic moiety with the upper and bottom part of the active site.

**FIGURE 3:**
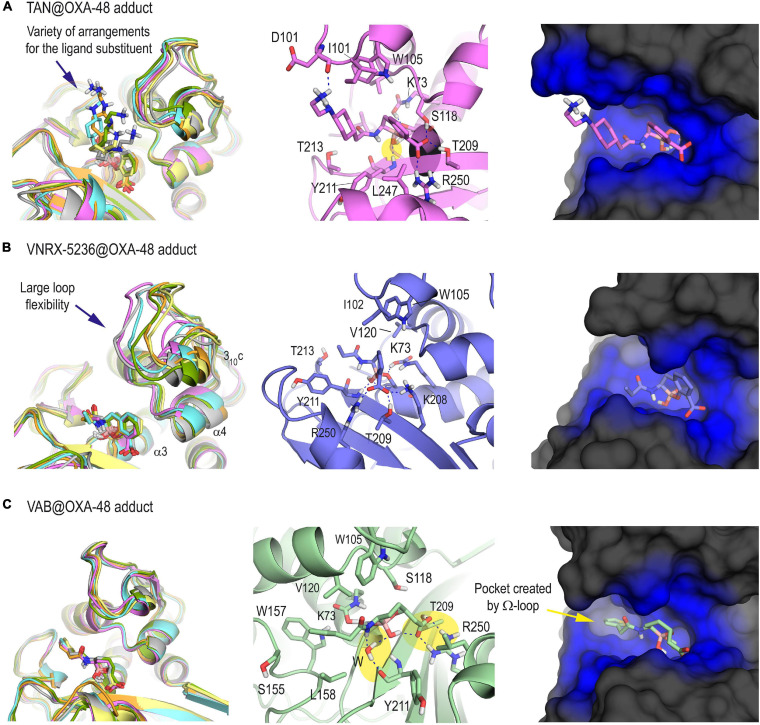
3D structure of OXA-48 enzyme inactivated by taniborbactam **(A)**, VNRX-5636 **(B)**, and vaborbactam **(C)** obtained by MD simulation studies. For each panel, the superposition of several snapshots from the 100 ns of simulation, the interactions of the modified ligand and the overall overview are shown. For the latter case, snapshots taken after 90 ns of simulation are shown. Note the large variety of arrangements of the amide moiety of the modified taniborbactam, as well as the weak interaction of the active site-covering loop involving helix 3_10_c with the modified VNRX-5236. See how the boronate moiety of the modified vaborbactam is anchored in the active site via a water molecule, while for all the bicycle boronate derivatives studied herein direct contacts with main carbonyl group of Y211 are observed (yellow shadow). Relevant side chain residues are shown and labeled. Key hydrogen bonding and electrostatic interactions are shown as blue dashed lines. The enzyme area surrounding the adduct is highlighted in blue.

Once the methodology employed was validated and the protonation state of the key residues in the catalysis was determined, the inactivation of the OXA-48 enzyme by taniborbactam, vaborbactam, and VNRX-5236 was studied *in silico* for which 3D structural information is not available. VNRX-5236 was included in this study to understand the impact of the amide moiety in taniborbactam inhibition. Thus, following a similar procedure as for QPX7728@OXA-48 adduct, the three enzyme adducts were constructed from the proposed arrangements obtained by molecular docking and then subjected to 100 ns of MD simulation. As expected, the anchoring of the central core of taniborbactam and VNRX-5236 to the enzyme active site resulted to be similar, and the main differences reside in the arrangement of the group adjacent to the boron atom. For taniborbactam, a variety of arrangements of the amide moiety were identified during the simulation because this part of the compound, which showed to adopt an axial disposition, is quite exposed to the solvent environment (Figure 3A). Despite this, the six-membered ring of the amide substituent would interact with the side chain residues I102 and/or W105, which are located on the loop that connects helix α3 and α4, and during some parts of the simulation, the amino group of the amide substituent would interact with the residue D101, also placed on this loop. On the contrary, for VNRX-5236, the position of its substituent showed to be quite frozen, and a large flexibility of the loop was observed during simulation as no relevant interactions between the inner part of this loop and the amide substituent were observed (Figure 3B). All these facts make taniborbactam more surrounded by the enzyme than VNRX-5236. Moreover, the binding of the modified vaborbactam in the active site revealed to be different to the bicyclic boronate inhibitors herein studied because: (1) the amide moiety, which would adopt an equatorial disposition instead of axial, would interact with the pocket formed by the Ω-loop in which W157 is located; (2) the interaction of the carboxylate moiety seems to be different from the other inhibitors and would only involve the residues R250 and T209; and more importantly (3) the boronate moiety would be anchored in the active site via a water molecule, while for all the bicycle boronate derivatives studied herein direct contacts with main carbonyl group of residue Y211 were observed (Figure 3C). The lower number and strength of direct polar contacts with the enzyme seems to be responsible of the weaker inhibitory potency of vaborbactam against OXA-48 (Table 1).

Overall, our computational studies revealed that the most remarkable difference in the binding of QPX7728 compared with the structurally related bicyclic boronate inhibitors taniborbactam and VNRX-5236 would be the favorable interactions achieved by its horizontal arrangement in the active site. This disposition seems to be induced by the presence of the cyclopropane ring on the opposite face of the six-membered boronate ring and the lack of a flexible substituent in the opposite face. A similar arrangement could not be achieved by taniborbactam and VNRX-5236 since the flexible amide moiety proved to adopt an axial conformation that prevent it, and as a result, these ligands would be less wrapped by the enzyme. For vaborbactam, the higher flexibility of the cyclic boronate moiety would facilitate the equatorial disposition of the substituent, which would be embedded by the enzyme in the pocket created by the Ω-loop. However, the latter arrangement would prevent from maximizing direct contacts with the enzyme.

#### OXA-23 and OXA-24/40 Enzymes

Following a similar approach as for the OXA-48 adducts, the inactivation of OXA-23 and OXA-24/40 by QPX7728, taniborbactam, VNRX-5236, and vaborbactam was studied *in silico*. For these inhibitors, no 3D structural information of the corresponding enzyme adducts is available. It has been reported that OXA-23 and OXA-24/40 enzymes are characterized by an uncommon architecture of the active site, consisting in a highly hydrophobic tunnel-like entrance mainly achieved by the side chains of two residues, a phenylalanine (OXA-23, F110) or a tyrosine (OXA-24/40, Y112) and a methionine [M221 (OXA-23), M223 (OXA-24/40)] (Lahiri et al., 2015). By this channel, the enzyme restricts the entry to only certain substrates, and enhances its catalytic efficiency by providing additional stabilizing interactions through the inner part of the hydrophobic bridge, thus fixing the substrates in the optimal conformation for hydrolysis (Stewart et al., 2019). The analysis of the intrinsic shape-changing motions of the unbound forms of OXA-23 and OXA-24/40 enzymes during 100 ns of simulation highlighted that: (1) the bridge-like structure of both enzymes is also observed in the free form; and (2) for OXA-24/40, a more closed arrangement of the active site is achieved since the hydrophobic bridge also involves the side chain of the conserved residue W221 (Supplementary Figure 2). Furthermore, examination of the vibrational modes for both enzymes in the unbound form calculated by principal-component analysis as implemented in AMBER, revealed significant differences in the plasticity of the upper part of the bridge in which Y112 (OXA-24/40) and F110 (OXA-23) are located (Supplementary Figure 3). Thus, while for OXA-23, this region of the enzyme is flexible, a more reduced plasticity was obtained for OXA-24/40. Altogether, these findings suggested that for the OXA-24/40 enzyme the overall architecture of the tunnel-like entrance is more rigid than for OXA-23, and the changes induced by ligand when binding would be mainly located in the bottom part of this structural motif, thus involving residues M223 and W221.

**FIGURE 4:**
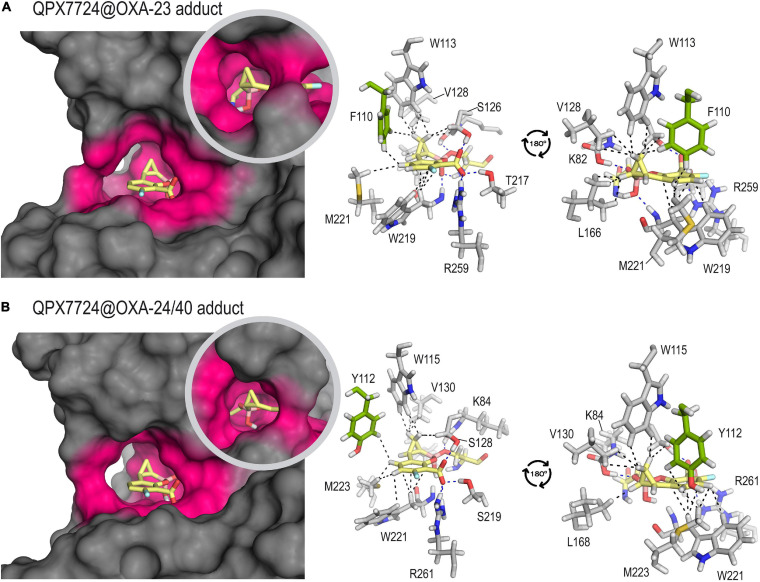
Inactivation of OXA-23 **(A)** and OXA-24/40 **(B)** by QPX7728 obtained by MD simulation studies. Overall and detailed views are provided. Snapshots taken after 90 ns of simulation are shown. Relevant side chain residues are shown and labeled. The enzyme area surrounding the adduct is highlighted in pink. Key polar (hydrogen bonding and electrostatic, blue) and CH-π and apolar interactions (black) are shown as dashed lines. Note the high amino acid sequence similarity of the active site residues in close contact with the modified inhibitor for both adducts. The main sequence difference is residue F110 (OXA-23) that is replaced by Y112 in OXA-24/40, both showed in green.

The results of our computational studies on the QPX7728@OXA-23 and QPX7728@OXA-24/40 enzyme adducts showed that the ability of this highly conformationally restricted bicyclic boronate inhibitor to lay into the active site would also be responsible of its high inhibitory capacity (Figure 4). Thus, for both enzymes, the modified QPX7728 would be embedded in the active site, which would be surrounded by the hydrophobic bridge. For both adducts, the overall structure of the complex would be very stable as no significant changes were identified either in the modified ligand as in the active site architecture during simulation (Supplementary Figure 4). As a result, the lipophilicity of the active site would be increased, thus avoiding the entrance of the water molecule for adduct hydrolysis and turnover. Under this arrangement, numerous apolar and CH-π interactions between both faces of the aromatic ring, as well as the cyclopropyl moiety of the ligand with the residues on the vicinity were identified, specifically residues W113/W115, M221/M223, V128/V130, L166/L168, W219/W221, F110/Y112, and S126/S128 (carbon side chain) in OXA-23 and OXA-24/40, respectively. Although the K_i_ or IC_50_ values of QPX7728 against OXA-24/40 has not been reported, considering its similarity with OXA-23 either on the amino acid sequence of its active site, as well as on the resulting architecture of the adduct, it is expected that the inhibitory capacity of QPX7728 against this enzyme would be also excellent.

**FIGURE 5:**
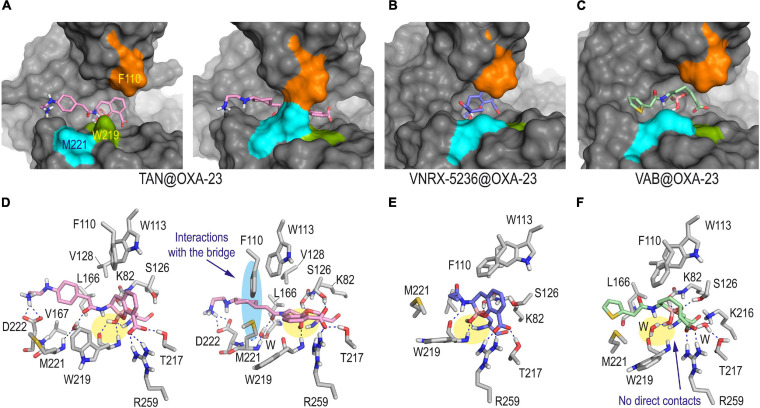
Overall and detailed view of the covalent modification of OXA-23 by taniborbactam **(A,D)**, VNRX-5236 **(B,E)**, and vaborbactam **(C,F)** obtained by MD simulation studies. The residues involved in the tunnel-like entrance F110 (orange) and M221 (cyan) and the proximal conserved residue W219 (green) are highlighted in the surface representations. Relevant side chain residues are shown and labeled. Hydrogen bonding interactions are shown as blue dashed lines. For TAN@OXA-23 adduct, the two main poses observed during simulation in a 1:1 ratio are provided: one showing an open active site (snapshot @40 ns) and the second exhibiting the usual tunnel-like architecture (snapshot @80 ns). For the other adducts, snapshots taken after 90 ns of simulation are shown. The contacts of the boronate moiety are highlighted with a yellow shadow. Note how only taniborbactam would be partially surrounded by the hydrophobic bridge (highlighted with a blue shadow) when horizontal arrangement was achieved. Vaborbactam would be the only inhibitor that would not establish direct contacts with the main backbone of OXA-23. TAN, taniborbactam; VAB, vaborbactam.

The simulation studies carried out with OXA-23 and OXA-24/40 enzymes covalently modified by taniborbactam, VNRX-5236 and vaborbactam predicted a completely distinct scenario to the above mentioned for QPX7728. Thus, for the taniborbactam@OXA-23 adduct, two main arrangements were identified in a 1:1 ratio, which differ mainly in the disposition of the bicyclic ring (Figures 5A,D). Thus, one of them would be close to that observed for QPX7728, thus with the ring lied out over the bottom of the active site and more surrounded by the hydrophobic bridge, while for the second pose, the aromatic ring would be rotated ∼60° (vertical disposition), affording a more open conformation of the active site. For the latter pose, the aromatic core of the modified ligand would interact with the aromatic rings of F110 and W113, which are involved in the upper inner part of the active site-covering loop. For both poses, distinct arrangements of the amide substituent in the modified taniborbactam were identified, but in all of them the terminal amino group would establish a strong electrostatic interaction with the carboxylate group of D222, which is located close to M221 (Figure 5D and Supplementary Figure 5). Although the modified taniborbactam can adopt a horizontal disposition during 50% of the simulation that would promote favorable hydrophobic interactions with the inner part of the bridge, these contacts would not be as numerous and strong as for QPX7728. Thus, the lack of the cyclopropane moiety and the presence of the amide group seems to prevent the covering of the enzyme achieved for QPX7728. For VNRX-5236@OXA-23 and vaborbactam@OXA-23 adducts, only vertical arrangements were observed, leading to more open conformations of the enzyme active site (Figures 5B,C,E,F and Supplementary Figure 5). Moreover, as for the OXA-48 enzyme, no direct contacts between the cyclic boronate moiety and the protein backbone were identified in the vaborbactam@OXA-23 adduct, which are observed in the other adducts.

**FIGURE 6:**
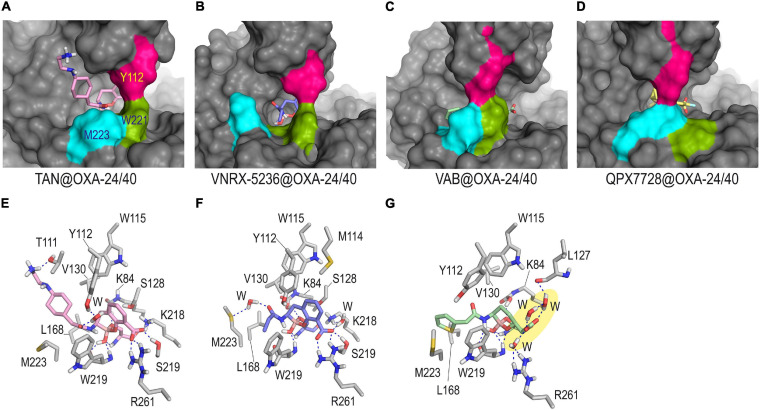
Overall and detailed view of the covalent modification of OXA-24/40 by taniborbactam **(A,E)**, VNRX-5236 **(B,F)**, and vaborbactam **(C,G)** obtained by MD simulation studies. Inactivation of OXA-24/40 by QPX7728 is also shown for comparison in panel **(D)**. The residues involved in the tunnel-like entrance, Y112 (pink), M223 (cyan), and W221 (green) are highlighted. Snapshots taken after 90 ns (TAN@OXA-24/40, QPX7728@OXA-24/40), and 80 ns (VNRX-5236@OXA-24/40, VAB5236@OXA-24/40) of simulation are shown. Relevant side chain residues are shown and labeled. Hydrogen bonding interactions are shown as blue dashed lines. Note how for TAN@OXA-24/40 and VNRX-5236@OXA-24/40 the extra hydrogen bonding interactions promoted by the amide group would induce significant modifications on the hydrophobic bridge architecture. No direct contacts between the carboxylate group of the modified vaborbactam and the enzyme residues were identified (yellow shadow). TAN, taniborbactam; VAB, vaborbactam.

The results of our computational studies on the inactivation of the OXA-24/40 enzyme by taniborbactam and VNRX-5236 predicted that these compounds: (1) would cause significant changes in the arrangement of the hydrophobic bridge of the enzyme, in order to react with the catalytic serine; and (2) would not provide a horizontal arrangement that would facilitate favorable interactions from both faces of the bicyclic moiety with the inner part of the bridge as observed for QPX7726 (Figure 6). All this seems to be caused by the amide substituent that would adopt an axial arrangement and would be partially fixed in the vicinity of the boronate core through hydrogen bonding interaction between the carbonyl group of its amide chain and the phenol group of Y122 or the thioether group of M223, in both cases mediated by a water molecule. It is important to bear in mind that the formation of the corresponding Michaelis complexes, which triggers the covalent modification of the catalytic serine, may also be compromised by the need to achieve less frequent arrangements of the tunnel-like entrance. For taniborbactam@OXA-24/40 adduct, the terminal end of the amide substituent would be flexible and different arrangements were identified during simulation (Supplementary Figure 5). For vaborbactam@OXA-24/40 adduct, although the modified inhibitor would be surrounded by the bridge, the hydrogen bonding interactions involving the carboxylate group would be much weaker than for the bicyclic boronate inhibitors. Thus, no direct interactions between the carboxylate group of the ligand and the enzyme residues in the vicinity were identified during the simulation. Only contacts with the main carbonyl group of L127 through a network of water molecules were observed. It should be noted that a key recognition motif in the anchoring of the inhibitor to the active site is the carboxylate group present in all inhibitors, as well as carbapenems and cephalosporins. For OXA-24/40 enzyme, this is mainly performed by interaction with residues R261 and S219. Altogether, the pre-organization of the ligand conformation achieved through the introduction of the bicyclic boronate group seems to facilitate the interaction with an active center of limited induced-fit plasticity like OXA-24/40 and OXA-23 enzymes. However, the axial conformation of the amide group in taniborbactam needed to interact in such a rigid cavity, seems to penalize the binding.

### Interaction of QPX7728 and Taniborbactam Against Metallo-Carbapenemases (VIM-2, NDM-1, and IMP-1)

Another differentiating feature between QPX7728 and taniborbactam is their distinct selectivity between B1 enzymes (VIM-1/VIM-2, NDM-1, IMP-1). Unlike no significant differences are observed against VIM-2 and NDM-1 enzymes, as for both cases the inhibition is in the low nanomolar range, the same does not happen against IMP-1 (Table 1). Surprisingly, for IMP-1 enzyme, the inhibitory potency of QPX7728 is about fourfold higher than that of taniborbactam, when IC_50_ values are considered, and up to 130-fold higher if K_i_ values are compared. In any case, for both compounds the inhibition is in the low micromolar range, which are encouraging results considering the lack of efficient inhibitors against IMP-1. This experimentally observed selectivity of QPX7728 against the IMP-1 enzyme, as well as the lower efficacy of bicyclic boronate inhibitors against this type of B1 enzyme is intriguing. It is worth highlighting that no crystal structures of IMP-1 in complex with bicyclic boronate inhibitors have been reported so far, and therefore the molecular basis that justify this selectivity is still unknown.

**FIGURE 7:**
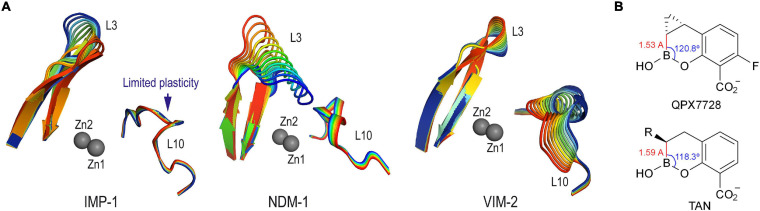
**(A)** Detailed view of the intrinsic induced-fit motion of the L3 and L10 loops of IMP-1, NDM-1 and VIM-2 enzymes obtained by examination of the vibrational modes. The main vibrational modes are shown. Zn(II) ions are shown as spheres. Note how IMP-1 is the enzyme in which the loop that surrounds the two metal centers (L10) has the least plasticity. **(B)** Relevant carbon-boron distances and carbon-boron-oxygen dihedral angles in QPX7728 and taniborbactam.

The available crystal structures of the IMP-1 enzyme, which are mainly in the unbound form and in complex with thiol derivatives, showed that the two metal centers are much closer to each other than in the VIM-2 and NDM-1 enzyme complexes. Therefore, to achieve enzyme complexes as observed in the crystal structures of QPX7728@VIM-2 (PDB ID 6V1P) (Hecker et al., 2020), taniborbactam@VIM-2 (PDB ID 6SP7) (Liu et al., 2020), QPX7728@NMD-1 (PDB ID 6V1M) (Hecker et al., 2020), and taniborbactam@NMD-1 (PDB ID 6RMF) (Krajnc et al., 2019), both Zn(II) ions must be spaced apart to accommodate several atoms between the two metal nuclei. Accordingly, the enzyme loop that surrounds both ions (bottom of the active site), namely L10, needs to have some flexibility to facilitate the distancing. We have shown recently that while the active site lid, namely L3 loop, is quite flexible for the three enzymes, IMP-1, NDM-1, and VIM-2, significant differences were identified in the bottom of their active sites (Figure 7A; Lence and González-Bello, 2021). Thus, IMP-1 showed to be the enzyme with the least flexible L10 loop of the three, as barely any motion of this area was observed during simulation. Therefore, the bottom of the active site of the IMP-1 enzyme seems to have reduced capacity to modulate its conformation to host compounds of distinct geometries, arrangements, and sizes. For conformationally constrained compounds as the bicyclic boronate inhibitors QPX7728 and taniborbactam, this feature could make the binding to IMP-1 difficult, and it is perhaps one of the reasons for their lower affinity against this enzyme compared with NDM-1 and VIM-2. Among both inhibitors, the introduction of a cyclopropane moiety in the adjacent carbon to the boronate moiety in QPX7728 modifies the geometry of the functional groups interacting with the two metallic nuclei. Thus, the calculated carbon-boron distance is reduced from 1.59 to 1.53 Å, and the carbon-boron-oxygen angle is increased from 118.3° to 120.8° in taniborbactam and QPX7728, respectively (Figure 7B). The latter geometrical changes might also contribute to the experimentally observed selectivity toward QPX7728. To explore the molecular basis of this selectivity, computational models of both enzyme complexes, as well as the corresponding Michaelis complexes, were constructed and compared. These studies were performed using the enzyme coordinates obtained in PDB ID 1VGN (2.63 Å, chain A), irreversibly inhibited by 3-(3-mercaptopropionylsulfanyl)propionic acid pentafluorophenyl ester (Kurosaki et al., 2005), because it is the crystal structure of IMP-1 enzyme showing the two Zn(II) ions more distanced.

**FIGURE 8:**
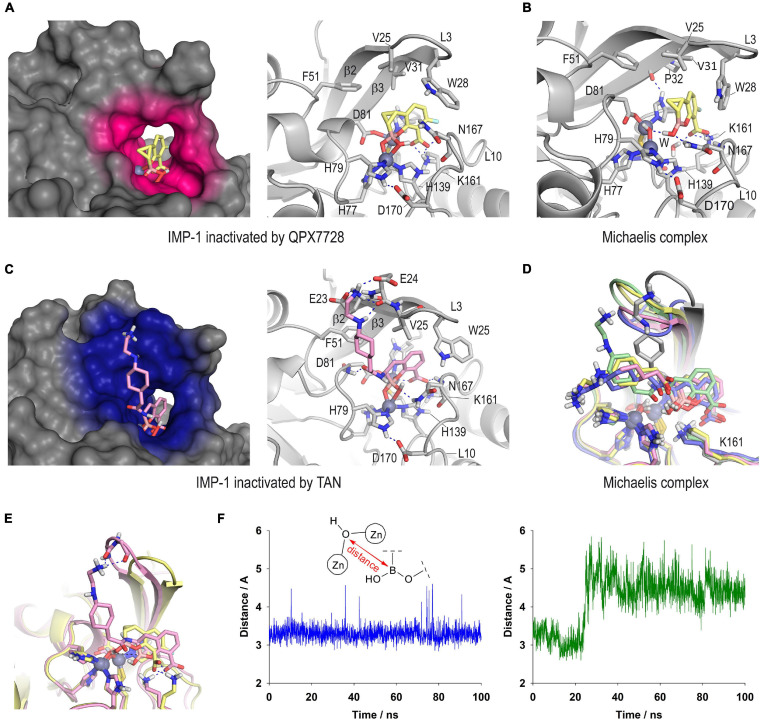
Proposed three-dimensional structure of IMP-1 inactivated by QPX7728 **(A)** and taniborbactam **(C)**. Detailed and overall views are shown. The enzyme area in close contact with the modified inhibitor is highlighted in pink and blue colors, respectively. Snapshots after 80 ns of simulation are shown. Relevant side chain residues are shown and labeled. Zn(II) ions are shown as spheres. **(B)** QPX7728@IMP-1 Michaelis complex (snapshot @80 ns). **(D)** Superposition of several snapshots [20 ns (gray), 40 ns (yellow), 60 ns (pink), 80 ns (green), 100 ns (blue)] from the 100 ns of simulation on taniborbactam@IMP-1 Michaelis complex. **(E)** Comparison of QPX7728@IMP-1 and taniborbactam@IMP-1 Michaelis complexes (snapshots @80 ns). **(F)** Variation of the relative distance between the oxygen atom of the nucleophilic hydroxyl group coordinated to the Zn(II) ions and the boron atom of QPX7728 (blue line) and taniborbactam (green line) in the corresponding QPX7728@IMP-1 and taniborbactam@IMP-1 Michaelis complexes during the simulation.

The outcomes of the computational studies carried out on the inactivation of the IMP-1 enzyme by QPX7728 and taniborbactam revealed no significant differences in the binding mode of both inhibitors relative to the bicyclic boronate moiety (Figure 8). Thus, for both cases, the bicycle would be embedded in the active site, and surrounded by the L10 loop, giving rise to closed complexes. The presence in the L3 loop of a tryptophan residue (W28), instead of a phenylalanine as for VIM-2 (F61) and NDM-1 (F70) further facilitates the sealing. The carboxylate group of the inhibitors would be anchored to the active site by an electrostatic interaction with the ε-amino group of K161, a hydrogen bond with the NH main chain amide of residue N167, as well as by coordination with the Zn2 site, which is observed in all the complexes of these types of compounds with other metallo-β-lactamases. The OH group in the boronate moiety would interact by hydrogen bonding with the side chain amide group of N167, although diverse arrangements of this residue were identified during simulation. Furthermore, the bicyclic boronate group would also establish numerous favorable apolar interactions with the side chains of the apolar residues of the inner part of the L3 loop and the β2 and β3 sheets, specifically W28, V25 and V31, in addition to F51. The main differences between the two inhibitors would lie in the extra binding interactions induced by the flexible amide substituent in taniborbactam, and the cyclopropane moiety in QPX7728. Regarding the amide moiety, unlike what is observed in the taniborbactam@VIM-2 complex (PDB ID 6SP7) in which this group is fixed at the entrance of the active site, a vertical arrangement was observed for taniborbactam@IMP-1. Thus, the terminal amino group of the amide moiety would interact by hydrogen bonding with the residues E23 and E24 located on the sheets β2 and β3, respectively, and its amide group would establish a hydrogen bond with the NH main chain amide of residue D81 (Figure 8 and Supplementary Figure 6). These polar interactions would be particularly strong since it appears captured very efficiently by these two negatively charged groups (Supplementary Figure 7). At the same time, the cyclopropane moiety in QPX7728 would be in close contact with the inner part of the active site, specifically by interacting with the phenyl group of F70, as well as the carbon chain of residue D81.

Altogether, the differences identified in the computational models of taniborbactam@IMP-1 and QPX7728@IMP-1 complexes do not seem to justify the higher selectivity of QPX7728 against this enzyme, at least regarding the resulting complex is concerned. The reasons are more likely due, in addition to the geometric changes resulting from the introduction of an extra constrained ring adjacent to the boronate core, to the Michaelis complex formation step that triggers the coordination to the di-Zn(II) system, leading to the inhibition. For the QPX7728@IMP-1 Michaelis complex, the bicyclic boronate moiety in QPX7728 would be located close to the Zn(II)-bound hydroxide anion, showing a very stable arrangement during the whole simulation (Figure 8B vs. Supplementary Figure 8). On the contrary, for the taniborbactam@IMP-1 Michaelis complex, the inhibitor is further away from the reactive center and its position is less frozen in the active site (Figures 8D,E). Moreover, the analysis of the variation of the relative distance between the oxygen atom of the nucleophilic hydroxyl group coordinated to the Zn(II) ions and the boron atom of the inhibitors showed that it varied between 2.9 and 3.6 Å (average of 3.3 Å) for QPX7728@IMP-1 and between 4.0 Å and 5.5 Å (average of 4.5 Å) for taniborbactam@IMP-1, considering the last 70 ns of simulation (Figure 8F). As for serine-β-lactamases, the sp2 form of the inhibitors proved to be the more stable than the corresponding sp3 form (Supplementary Figure 9). Therefore, these outcomes suggested that the rigidification of the bicyclic boronate group conformation through the introduction of additional conformational constraints (cyclopropane) in the adjacent carbon to the boron atom, may facilitate a more optimal geometry of the ligand for the formation of the adduct with the IMP-1 enzyme, which is the one with the least flexible active site among the B1 enzymes.

## Conclusion

In summary, the results of the computational studies carried out with the carbapenem hydrolyzing class D β-lactamases OXA-48, OXA-23, and OXA-24/40 revealed that the most differentiating feature in the binding of QPX7728 compared with the structurally related bicyclic boronate inhibitors taniborbactam and VNRX-5636 would be its horizontal arrangement in the active site. This disposition, which seems to be induced by the presence of the cyclopropane ring and the lack of a flexible amide substituent in the opposite face, would promote a set of favorable apolar and CH-π interactions with residues located on top and bottom of the active site, leading to an adduct wrapped by the enzyme. This extra binding interactions from both faces of the bicycle moiety might be responsible of the outstanding inhibitory capacity of QPX7728 against this clinically relevant serine-carbapenemases. The formation of the serine adduct would be triggered by deprotonation of the catalytic serine residue by the essential carboxylated lysine residue, followed by nucleophilic attack of the serine residue to the boronate group in QPX7728 (sp2 form), which would promote a 45° turn on the overall disposition of the bicycle core leading to the horizontal arrangement experimentally observed. The axial arrangement adopted by the amide substituent in taniborbactam and VNRX-5636 upon binding these enzymes would prevent this favorable arrangement from being achieved.

On the other hand, the *in silico* studies carried out to shed light on the molecular basis of the differentiating selectivity of QPX7728 and taniborbactam against the IMP-1 enzyme suggested that would be due to differences on the Michaelis complex formation step. QPX7728 would bind to the IMP-1 active site in a more optimal arrangement for reaction with the Zn(II)-bound hydroxide anion, which leads to inhibition. The geometric changes in the boronate group resulting from the introduction of an extra constrained ring (cyclopropane) adjacent to the boron atom might also facilitate this binding, whose impact would be more pronounced for an enzyme such as IMP-1 characterized by its limited plasticity around the Zn(II) coordination region (L10 loop).

## Experimental Section

### Gaussian Minimization of the OXA-48 Active Site

The enzyme coordinates obtained in the crystal structure of OXA-48 from *K. pneumoniae* in complex with QPX7728 [PDB ID 6V1O (chain A)] were employed. For the minimization of the enzyme active site, the following amino acids were considered: side chains of I102, W105, S118, V120 (connected to S118 with V119 truncated as glycine residue), L158, and R251, all capped at Cα atoms as a methyl group; S70 and carboxylated K73 (connected through T71 and P72 both truncated as glycine residues) with acetyl and *N*-methyl caps; and K208, T209, G210, Y211 with acetyl and *N*-methyl caps. For the OXA-48@QPX7728 adduct studies, the enzyme coordinates in PDB ID 6V1O were used, while for the OXA-48@QPX7728 enzyme complex the coordinates obtained by docking were used (see below). These systems were minimized using the Gaussian 09W (Frisch et al., 2016) program package at density functional theory (DFT) level by means of the B3LYP functional, with the standard 6-31+G(d,p) (Petersson et al., 1988; Petersson and Al-Laham, 1991) basis set was used. Positional restraints to the Cα, Cβ, and methyl groups were used to preserve the structure of the active site.

### Ligand Preparation

Compounds QPX7728, taniborbactam, vaborbactam, and VNRX-5236 in their sp2 and sp3 forms were minimized using a restricted Hartree–Fock (RHF) method and a 6-31G(d) basis set, as implemented in the *ab initio* program Gaussian 09W. Partial charges were derived by quantum mechanical calculations by using Gaussian 09W, as implemented in the R.E.D. Server (version 3.0)^5^ (Dupradeau et al., 2010; Vanquelef et al., 2011), according to the RESP model (Cornell et al., 1995). The covalent modified catalytic serine residue by the inhibitors was parameterized using the R.E.D. server, by adding acetyl and *N*-methyl caps and applying the same procedure used for ligands. RESP charge calculation for the modified serine residue considered the net charge for both acetyl and *N*-methyl caps to be zero. The missing bonded and non-bonded interaction parameters were assigned, by analogy or through interpolation, from those already present in the general Amber force field (GAFF) database (Wang et al., 2004, 2006).

Boron parametrization was performed using the Seminario method (Seminario, 1996) implemented in the VFFDT (Zheng et al., 2016) and Paramfit programs (Kurt and Temel, 2020). Usual GAFF atom types were applied to all atoms except for boron. For the latter, two new boron atom types were defined: (1) b2 for a planar trigonal boron atom with sp2 hybridization; and (2) b3 for a tetrahedral boron atom with sp3 hybridization. To minimize the conformational effects, parameters involving b2 or b3 atoms were obtained as an average value for the different compounds. Additionally, both RHF/6-31G(d) and B3LYP/6-31G(d,p) levels of theory were employed in minimization and frequency calculations. Bond parameters for the oh-ho and os-c3 bonds, which are involved in the boronate core, were also calculated in the same manner, leading to similar values to those already present in the GAFF force field. Hence, for the latter bonds the available GAFF parameters were employed. Additional parameters for the boron atom were taken from Kurt and Temel (2020) and Tafi et al. (2005) works.

For metallo-β-lactamase studies, coordinates from the crystal structures of: (1) VIM-2 from *P. aeruginosa* in complex with QPX7728 (PDB ID 6V1P) and taniborbactam (PDB ID 6SP7); (2) NMD-1 from *K. pneumoniae* in complex with QPX7728 (PDB ID 6V1M) and taniborbactam (PDB ID 6RMF) were employed. MCPB.py (version 3.0)^6^ program of AmberTools 17 was used to obtain Zn(II) parameters, following the procedure describe in the Amber tutorial 20 (Li and Merz, 2016). Ligand parameters were obtained as described before. For ligands in the sp3 hybridization form coordinated to the Zn sphere, parameters were obtained considering only coordination to the Zn1 site, which is composed of three histidine residues and a hydroxide anion. The Seminario method was employed to generated force field parameters for the Zn(II) ions, at RHF/6-31G(d) level of theory with positional restraints to Cα and Cβ to keep an arrangement similar to the corresponding crystal structure. The Seminario/ChgModB method was used to perform the RESP charge fitting at the same level of theory used before, with only minimizing position of the hydrogen atoms. Average value for bond and angle constants were also employed. Using bond and angle values from crystal structures to generate the equilibrium values led to similar results. For IMP-1 enzyme, as no crystal structure in complex with ligands structurally related to the boron-based inhibitors herein studied, parameters obtained for VIM-2 enzyme complexes were employed.

### Docking Studies

They have been performed using GOLD 2020.2.0 (Jones et al., 1997) program and the protein coordinates from the crystal structures of: (1) VIM-2 from *P. aeruginosa* in complex with QPX7728 (PDB ID 6V1P) and taniborbactam (PDB ID 6SP7); (2) IMP-1 from *S. marcescens* (PDB ID 1VGN, chain A); (3) NMD-1 from *K. pneumoniae* in complex with QPX7728 (PDB ID 6V1M) and VNRX-5133 (PDB ID 6RMF); (4) OXA-48 from *K. pneumoniae* in complex with QPX7728 [PDB ID 6V1O (chain A)]; (5) OXA-23 from *A. baumannii* (PDB ID 4JF6) (Smith et al., 2013); (6) OXA-24/40 from *A. baumannii* (PDB ID 3FV7) (Bou et al., 2010). The experimental procedure used was similar to that described by us for 6-halopyridylmethylidene penicillin-based sulfone inhibitors (Vázquez-Ucha et al., 2021).

### Molecular Dynamics Simulation Studies

The ligand minimization, the generation, and minimization of inhibitor@enzyme adducts and binary complexes, and MD simulation of the resulting minimized adducts and complexes (100 ns) were carried following our previously described protocol (Vázquez-Ucha et al., 2021). The inhibitor@enzyme structures herein disclosed were created using the molecular graphics program PyMOL (DeLano, 2020). The stability and reliability of all the ligand@enzyme adducts and complexes herein described were verified by the analysis of the root-mean-square deviation (rmsd) for the whole protein backbone (Cα, C, N, and O atoms), the inhibitors and the modified ligand during the whole simulation by using the cpptraj module in AMBER 16 (Supplementary Figures S9–S14). Overall, relatively low rmsd values for the protein backbone were obtained. As expected for inhibitors having a flexible amide group, such as taniborbactam, VNRX-5236, and vaborbactam, higher rmsd values were observed.

### Vibrational Modes Calculations

The vibrational modes for IMP-1, VIM-2, NDM-1, OXA-23, and OXA-24/40 enzymes were calculated from the corresponding MD trajectories on the unbound forms by principal component analysis using the cpptraj module (Galindo-Murillo et al., 2015).

## Data Availability Statement

The raw data supporting the conclusions of this article will be made available by the authors, without undue reservation.

## Author Contributions

All authors listed have made a substantial, direct and intellectual contribution to the work, and approved it for publication.

## Conflict of Interest

The authors declare that the research was conducted in the absence of any commercial or financial relationships that could be construed as a potential conflict of interest.

## Publisher’s Note

All claims expressed in this article are solely those of the authors and do not necessarily represent those of their affiliated organizations, or those of the publisher, the editors and the reviewers. Any product that may be evaluated in this article, or claim that may be made by its manufacturer, is not guaranteed or endorsed by the publisher.

## Footnotes

1
https://clinicaltrials.gov/ct2/show/NCT03894046

2
https://clinicaltrials.gov/ct2/show/NCT03840148

3
https://clinicaltrials.gov/ct2/show/NCT04380207

4
https://clinicaltrials.gov/ct2/show/NCT04243863

5
http://upjv.q4md-forcefieldtools.org/RED/

6
https://ambermd.org/tutorials/advanced/tutorial20/mcpbpy.php
