# Supplementary material for: Molecular Basis of Bicyclic Boronate β-Lactamase Inhibitors of Ultrabroad Efficacy – Insights From Molecular Dynamics Simulation Studies
Source: Front Microbiol. 2021 Aug 4;12:721826. doi: 10.3389/fmicb.2021.721826 (PMC8371488; doi:10.3389/fmicb.2021.721826)
Supplement: Supplementary file 1 [file Data_Sheet_1.PDF]

Supplementary Material

Molecular Basis of Bicyclic Boronate β-Lactamase Inhibitors of Ultrabroad Efficacy ─ Insights from Molecular Dynamics Simulation Studies

Emilio Lence[1], Concepción González-Bello[1]*

[1]Centro Singular de Investigación en Química Biolóxica e Materiais Moleculares (CiQUS), Departamento de Química Orgánica, Universidade de Santiago de Compostela, Santiago de Compostela, Spain.

*E-mail: concepcion.gonzalez.bello@usc.es

Table of Contents

1. Figure S1……………..……………..….........................…..…..…………………….…….…….…....S2
2. Figure S2……………..……………..….........................…..…..…………………….…….…….…....S3
3. Figure S3………………..…………….........................…..…..…………………….…….…….…....S4
4. Figure S4………………..…………….........................…..…..…………………….…….…….…....S5
5. Figure S5……………..……………..….........................…..…..…………………….…….…….…....S6
6. Figure S6……………..…………...….........................…..…...…..……………………….…….…....S7
7. Figure S7……………………….....….........................…..…..……………….…………………….....S8
8. Figure S8……………………….....….........................…..…..……………….…………………….....S9
9. Figure S9…………..…..………....….........................…..……….……….……………..……....S10
10. Figure S10…………..…..…..……....….........................…..……….……….……………..……....S11
11. Figure S11…………..…..………....….........................…..……….……….……………..……......S12
12. Figure S12…………..…..………....….........................…..……….……….……………..……......S13
13. Figure S13…………..…..………....….........................…..……….……….……………..……......S14
14. Figure S14…………..…..………....….........................…..……….……….……………..……......S15

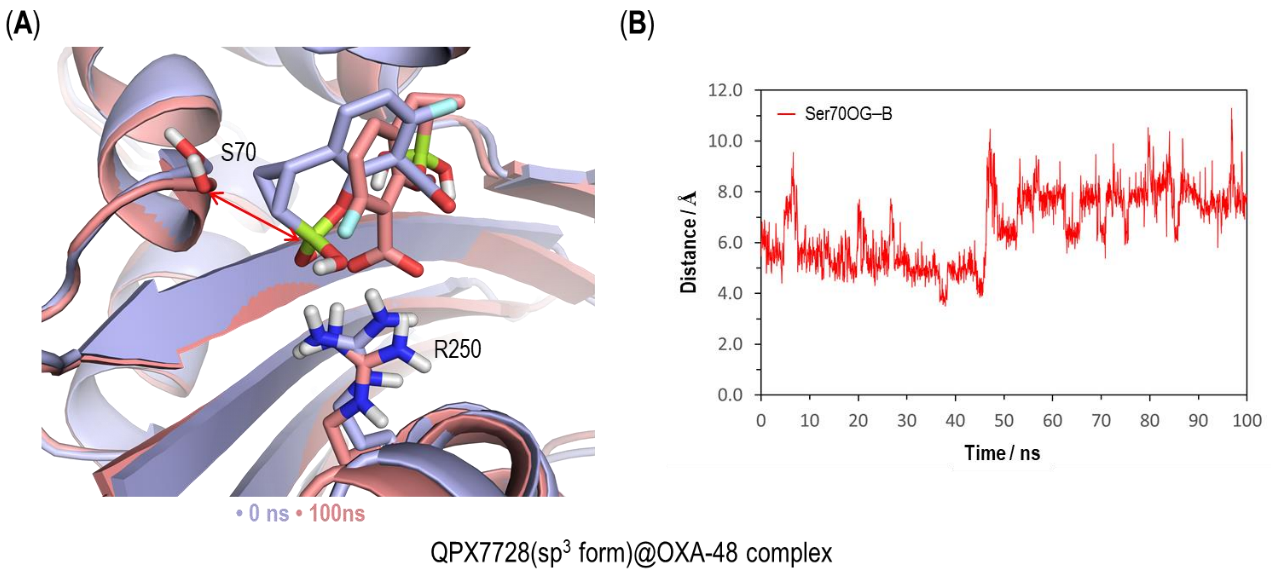

Figure S1. (**A**) Comparison of the binding mode of the sp3 form of QPX7728 at the beginning (0 ns), and after 100 ns of simulation in complex with OXA-48. The side chain of the catalytic S70 and R250 are shown and labeled. Boron atoms are highlighted in lime color. (**B**) Variation of the relative distance between S70 (O atom) and B atom of the ligand during the whole simulation. Note how the ligand rotates 180º during simulation to locate the negatively charged boronate moiety close to the guanidinium group of R250 and far from the nucleophilic S70.

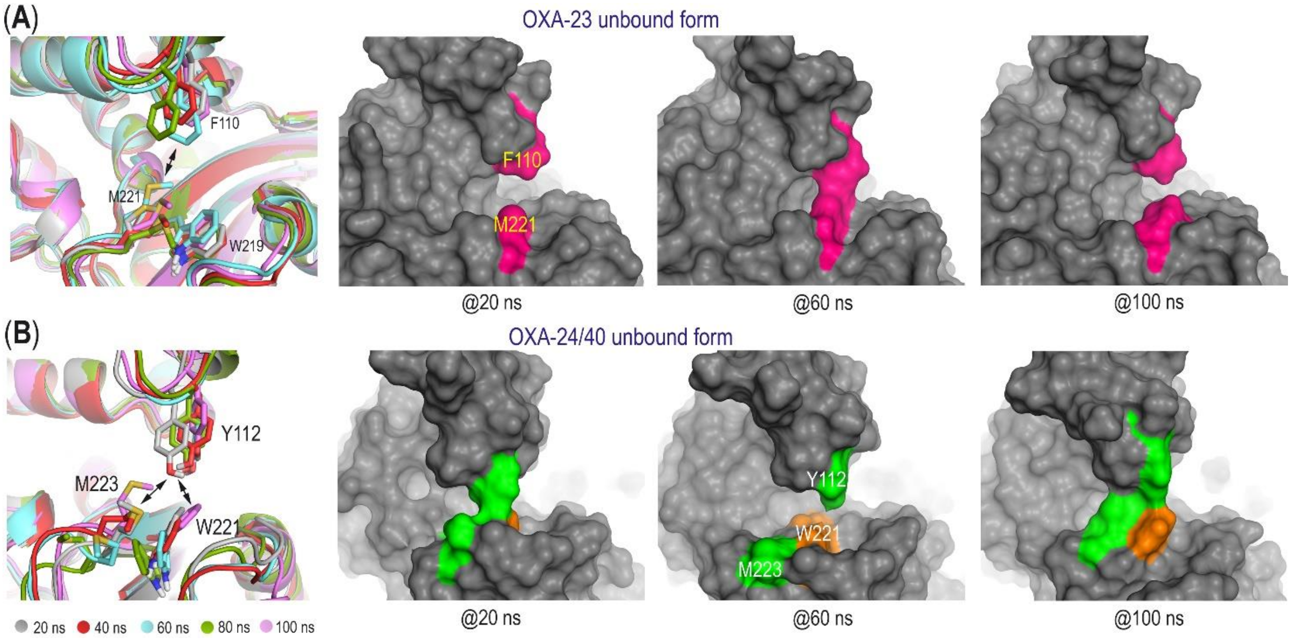

Figure S2. Intrinsic shape-changing motions of OXA-23 (**A**) and OXA-24/40 (**B**) enzymes obtained by MD simulation studies. For each panel, the superposition of several snapshots from the 100 ns of simulation, and the overall overview of the active site are shown. The side chain of the residues involved in the hydrophobic bridge are shown and their positions are highlighted in the surface representations. Note how for OXA-24/40 enzyme, the side chain of the conserved residue W221 is also involved in the hydrophobic bridge, leading to a less accessible active site.

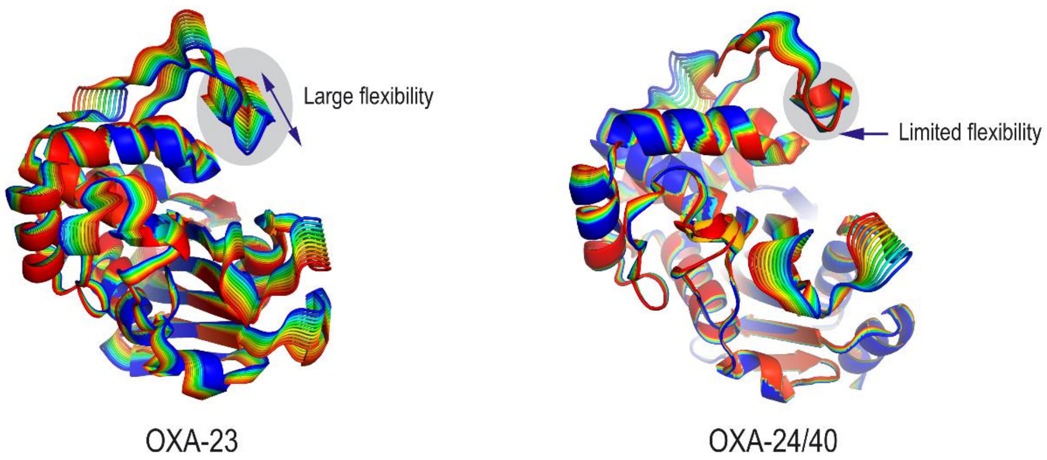

Figure S3. Overall view of the intrinsic shape-changing motion of OXA-23 and OXA-24/40 enzymes in the unbound form obtained by examination of the vibrational modes. The main vibrational modes are presented. Note how for OXA-24/40 the protein region involved in the hydrophobic bridge architecture of the active site, in which residue Y112 is allocated (gray shadow), is predicted to have limited flexibility.

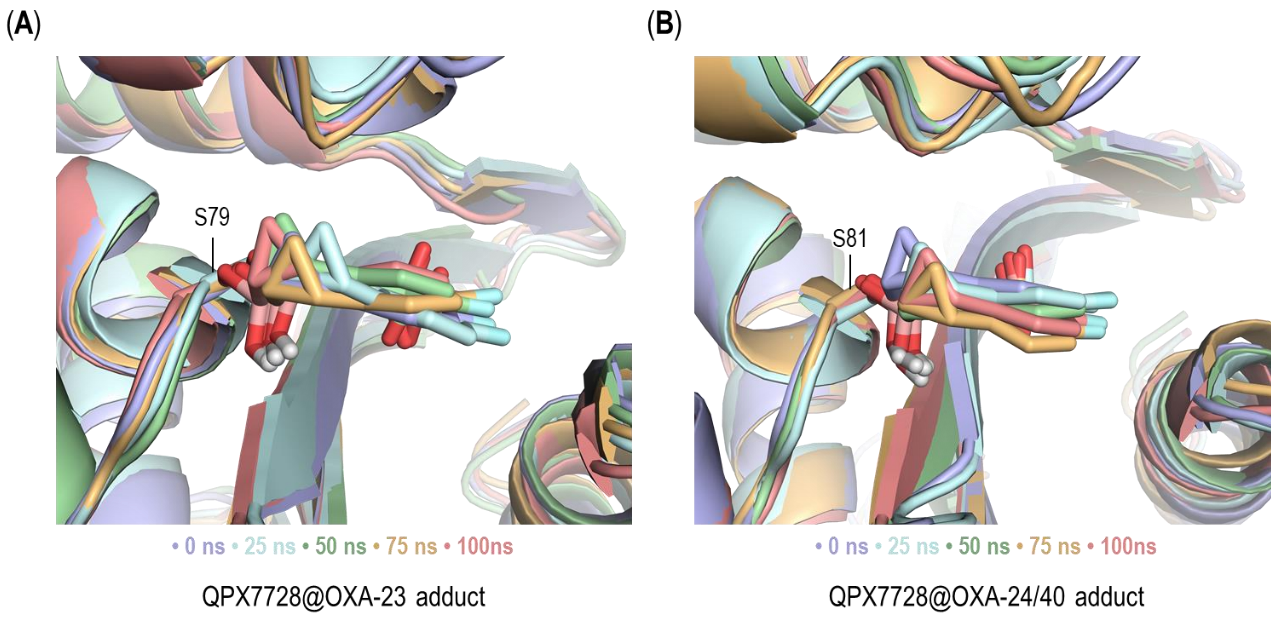

Figure S4. Superposition of several snapshots from the 100 ns of MD simulation on the QPX7728@OXA-23 (**A**) and QPX7728@OXA-24/40 (**B**) enzyme adducts. The modified catalytic serine residues are shown as sticks. Note how no significant changes are identified during simulation.

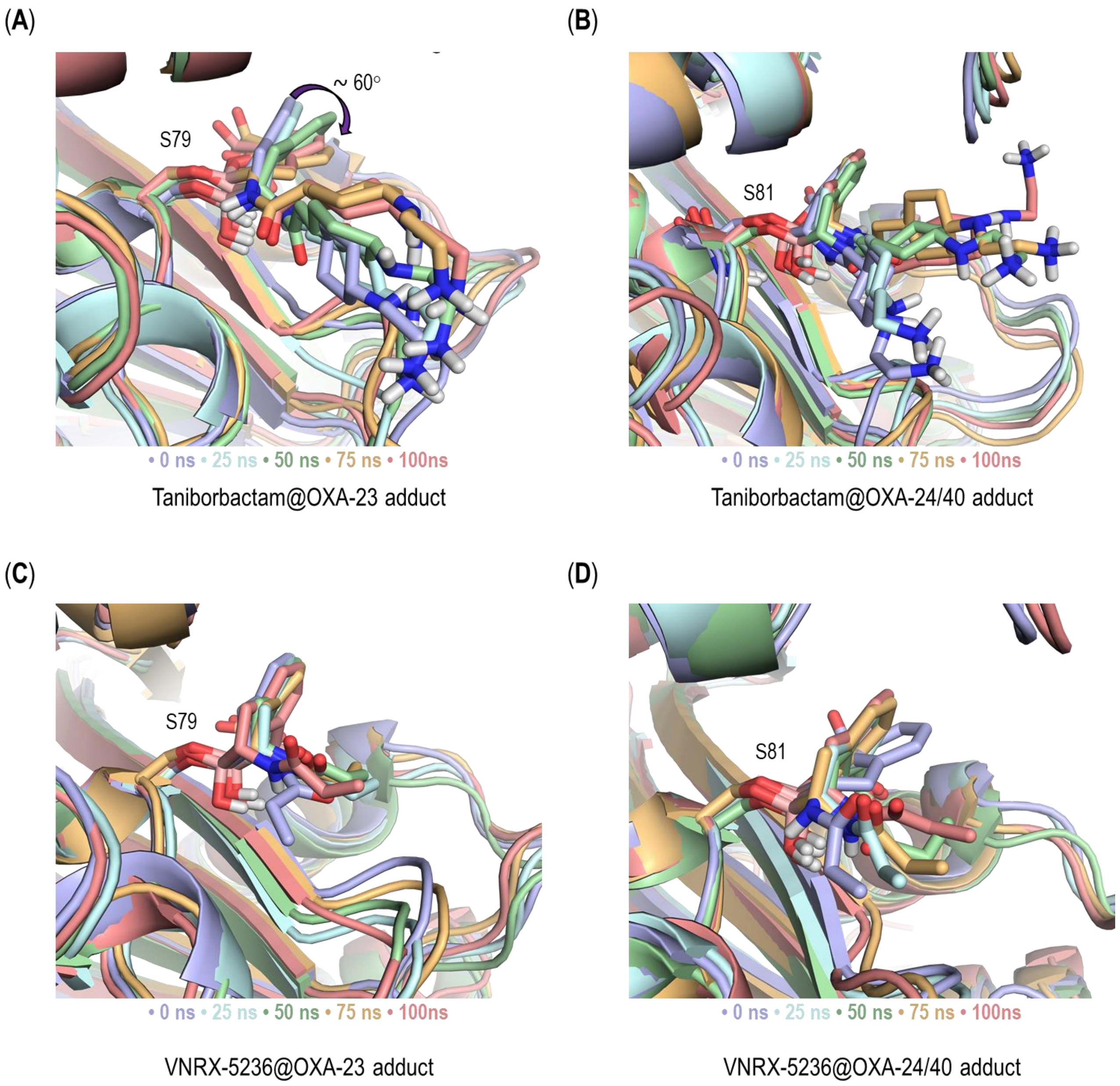

Figure S5. Superposition of several snapshots from the 100 ns of MD simulation on the taniborbactam@OXA-23 (**A**), taniborbactam @OXA-24/40 (**B**), VNRX-5236@OXA-23 (**C**), and VNRX-5236@OXA-24/40 (**D**) enzyme adducts. The modified catalytic serine residues are shown as sticks. Note how for all adducts significant changes in the amide side chain are identified during simulation. In some cases, several arrangements of the bicyclic boronate moieties are also observed.

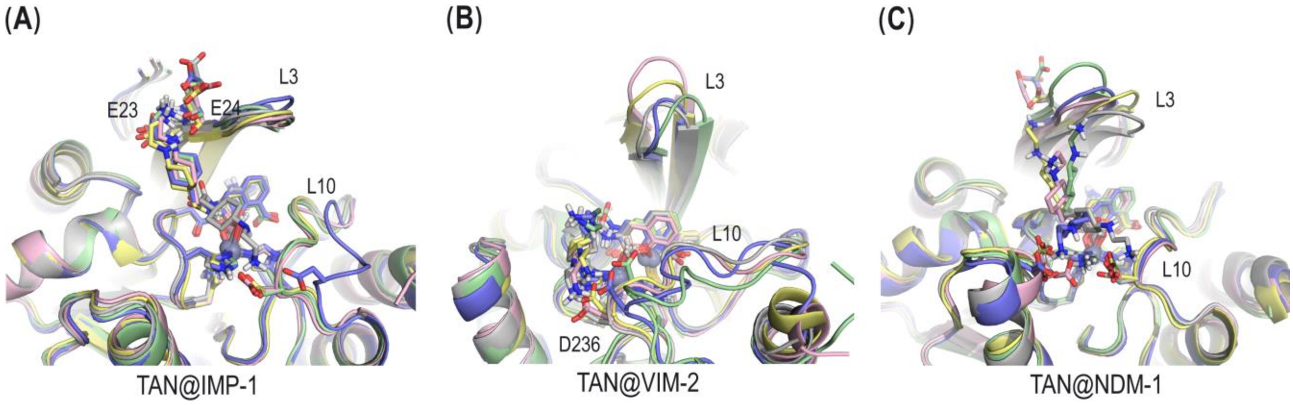

Figure S6. Superposition of several snapshots [20 ns (gray), 40 ns (yellow), 60 ns (pink), 80 ns (green), 100 ns (blue)] from the 100 ns of MD simulation on the: (**A**) taniborbactam@IMP-1; (**B**) taniborbactam@VIM-2; and (**C**) taniborbactam@NDM-1 complexes. The side chain residues coordinated to the two Zn(II) ions (spheres) and residues interacting with the terminal amino group are shown as sticks. Note how while for IMP-1 enzyme the side chain of taniborbactam would be interact mainly with the L3 loop (E23 and E24), for VIM-2 enzyme it would occur with the L10 loop (D236). For NDM-1 complex, the side chain of taniborbactam would be more flexible as a variety of arrangements were identified during simulation.

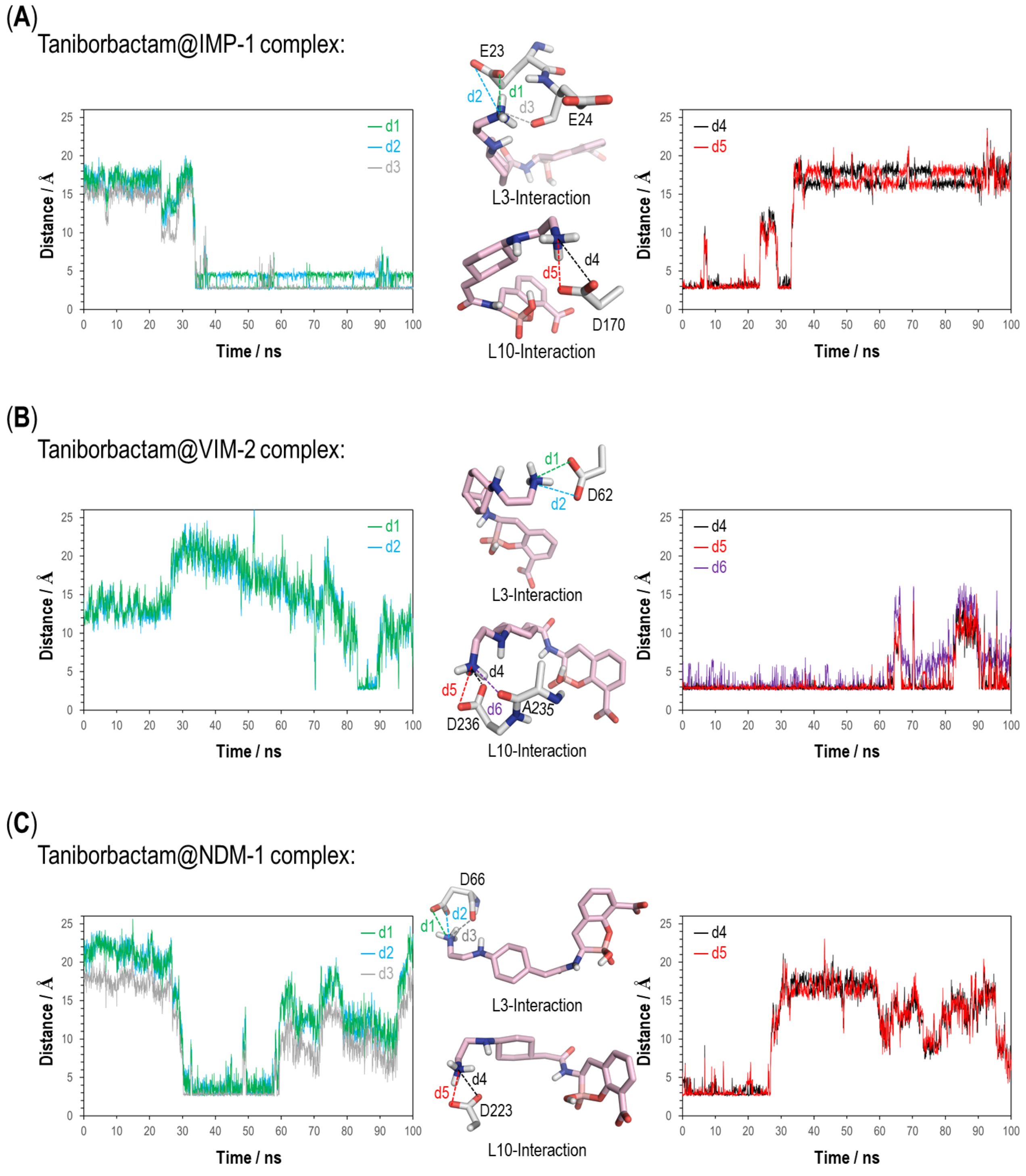

Figure S7. Variation of the relative distance between the ammonium group in taniborbactam with negatively charged residues present in L3 and L10 loops during 100 ns of MD simulations of the enzyme complexes: (**A**) taniborbactam@IMP-1; (**B**) taniborbactam@VIM-2; (**C**) taniborbactam@NDM-1. All distances were measured between ammonium N atom of taniborbactam and the following O atoms: (**A**) OE1, OE2 and carbonyl O atom of E23, and OD1 and OD2 O atoms of D170; (**B**) OD1 and OD2 of D62, OD1 and OD2 of D236, and carbonyl O atom of A235; (**C**) OD1, OD2 and carbonyl O atom of D66, OD1 and OD2 of D223.

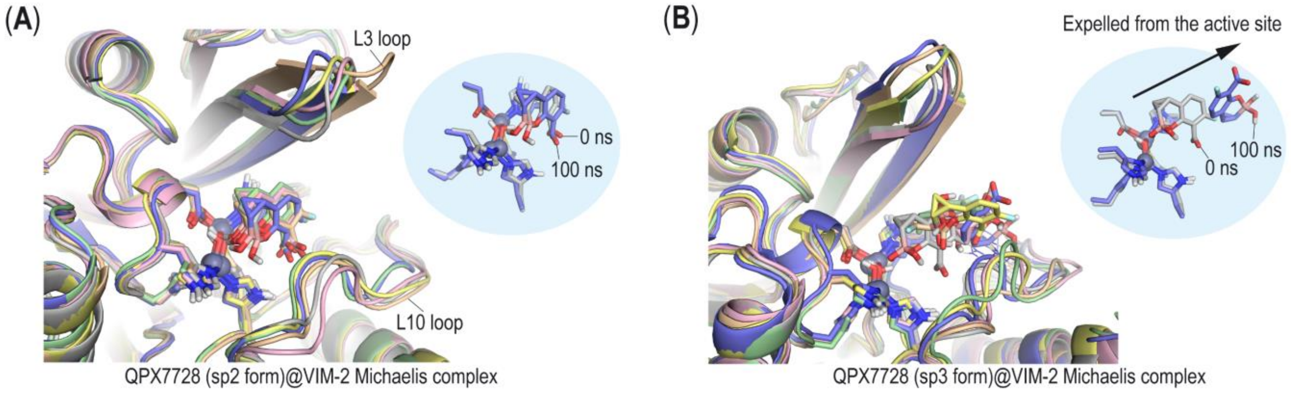

Figure S8. Superposition of several snapshots [0 ns (gray), 20 ns (wheat), 40 ns (yellow), 60 ns (pink), 80 ns (green), 100 ns (blue)] from the 100 ns of MD simulation on the QPX7728@VIM-2 Michaelis complex considering the sp2 (**A**) and sp3 (**B**) forms of the inhibitor. A detailed view of the start and end of the simulation is also provided in the blue circle. The side chain residues coordinated to the two Zn(II) ions (spheres) are shown as sticks. Note how the sp3 form of QPX7728 is not stable in the active site and expelled during the simulation.

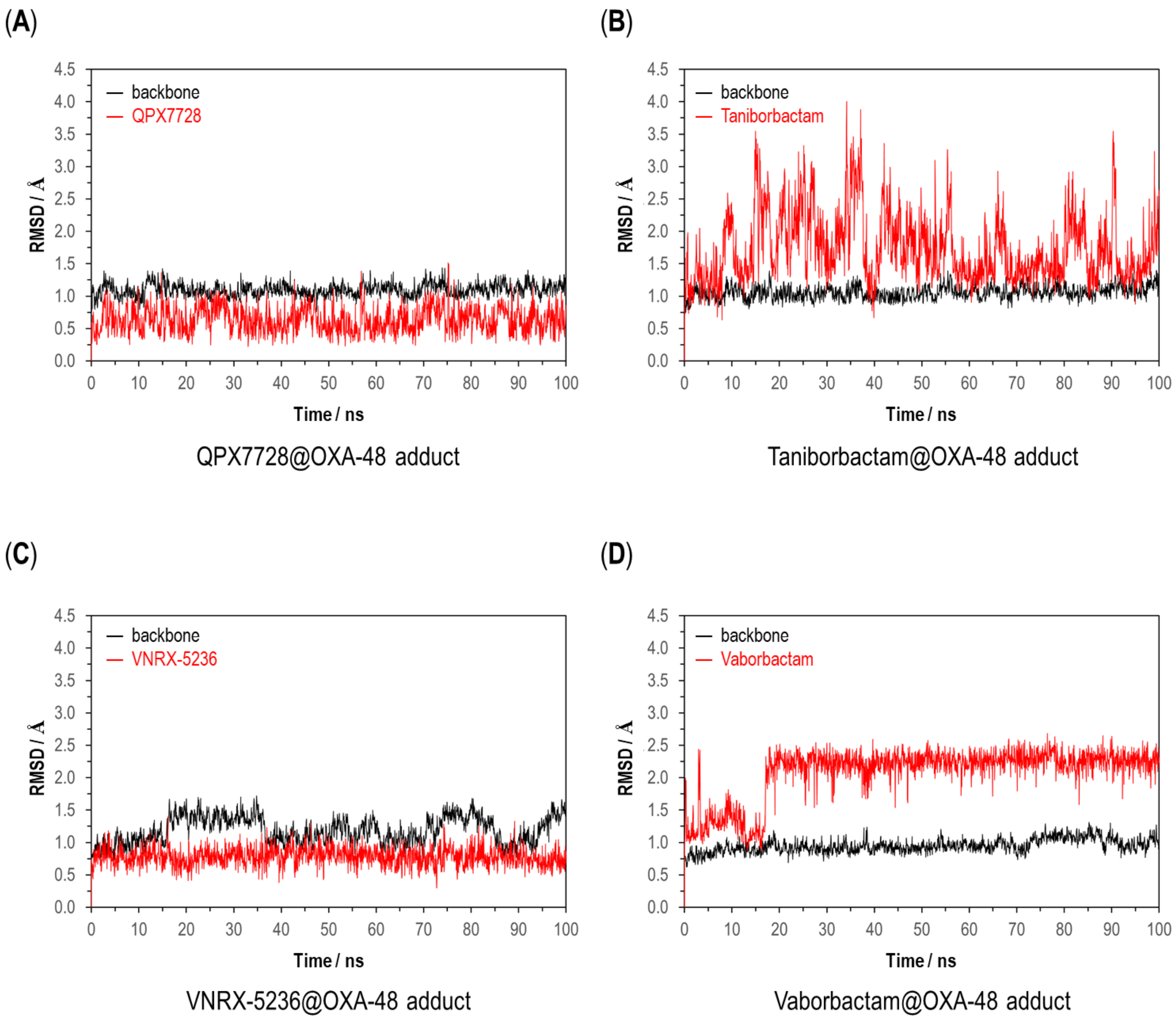

Figure S9. RMSD plots for the protein backbone (Cα, C, O and N atoms, black lines) and the modified ligands (heavy atoms, red lines) calculated from the MD simulations of the adducts: (**A**) QPX7728@OXA-48; (**B**) taniborbactam@OXA-48; (**C**) VNRX-5236@OXA-48; (**D**) vaborbactam@OXA-48.

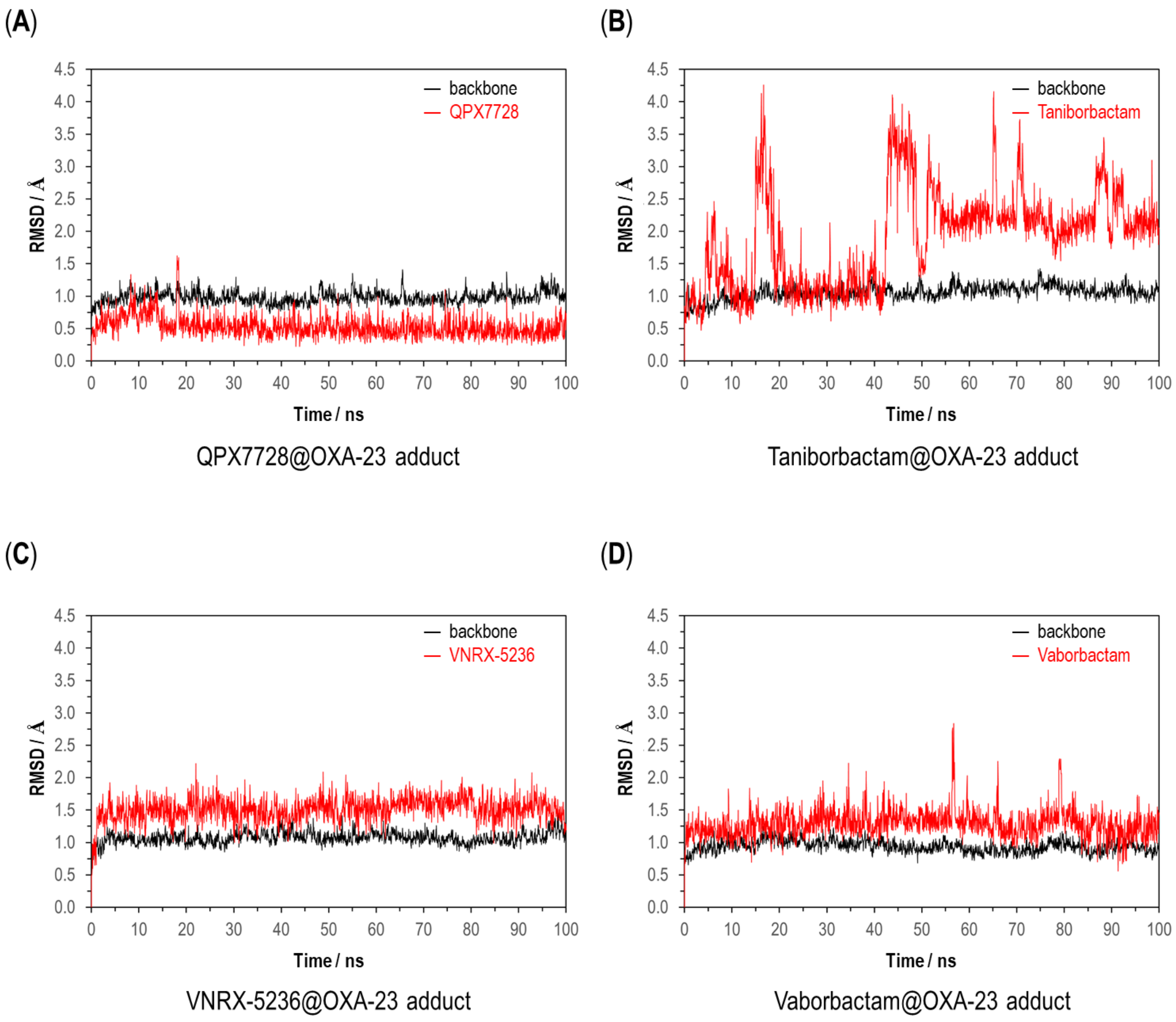

Figure S10. RMSD plots for the protein backbone (Cα, C, O and N atoms, black lines) and the modified ligands (heavy atoms, red lines) calculated from the MD simulations of the adducts: (**A**) QPX7728@OXA-23; (**B**) taniborbactam@OXA-23; (**C**) VNRX-5236@OXA-23; (**D**) vaborbactam@OXA-23.

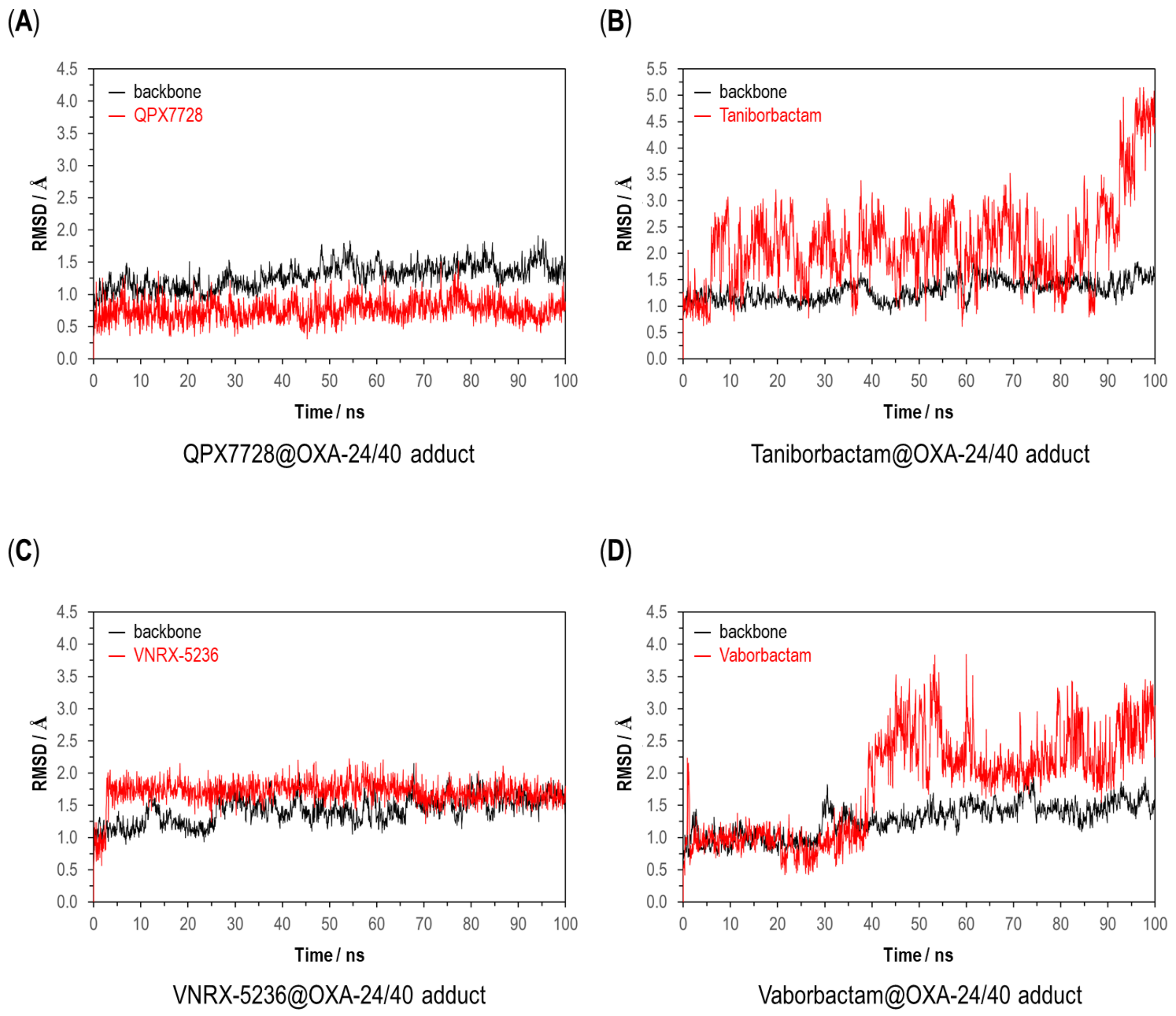

Figure S11. RMSD plots for the protein backbone (Cα, C, O and N atoms, black lines) and the modified ligands (heavy atoms, red lines) calculated from the MD simulations of the adducts: (**A**) QPX7728@OXA-24/40; (**B**) taniborbactam@OXA-24/40; (**C**) VNRX-5236@OXA-24/40; (**D**) vaborbactam@OXA-24/40.

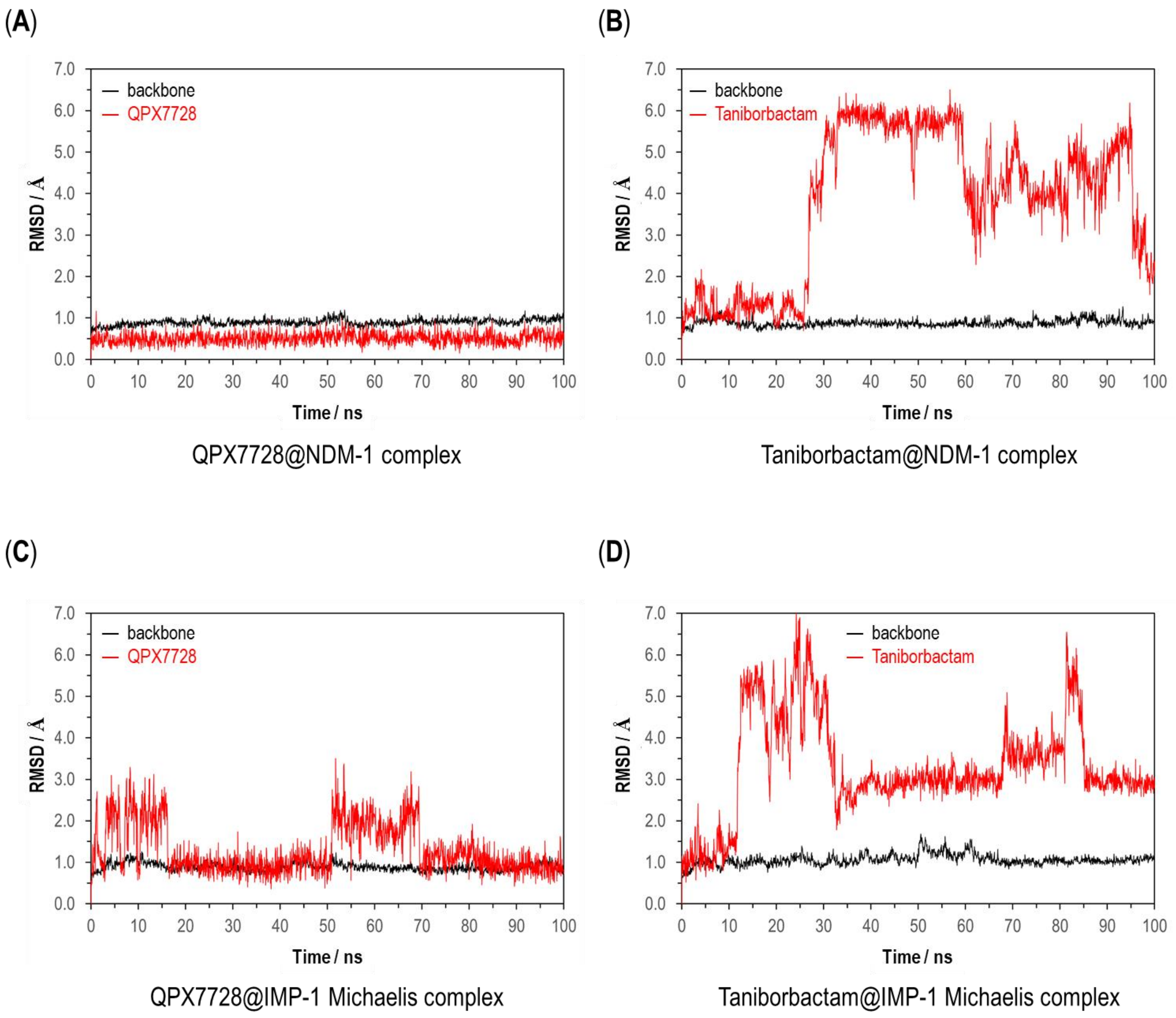

Figure S12. RMSD plots for the protein backbone (Cα, C, O and N atoms, black lines) and the modified ligands (heavy atoms, red lines) calculated from the MD simulations of the complexes: (**A**) QPX7728@IMP-1; (**B**) taniborbactam@IMP-1; (**C**) QPX7728@IMP-1 Michaelis complex; (**D**) taniborbactam@IMP-1 Michaelis complex. Note how QPX7728 is remarkably more stable than taniborbactam when binding the IMP-1 active site for Michaelis complex formation. The high rmsd values of the modified taniborbactam are due to the large variety of arrangements of its flexible amide moiety, which is absent in QPX7728.

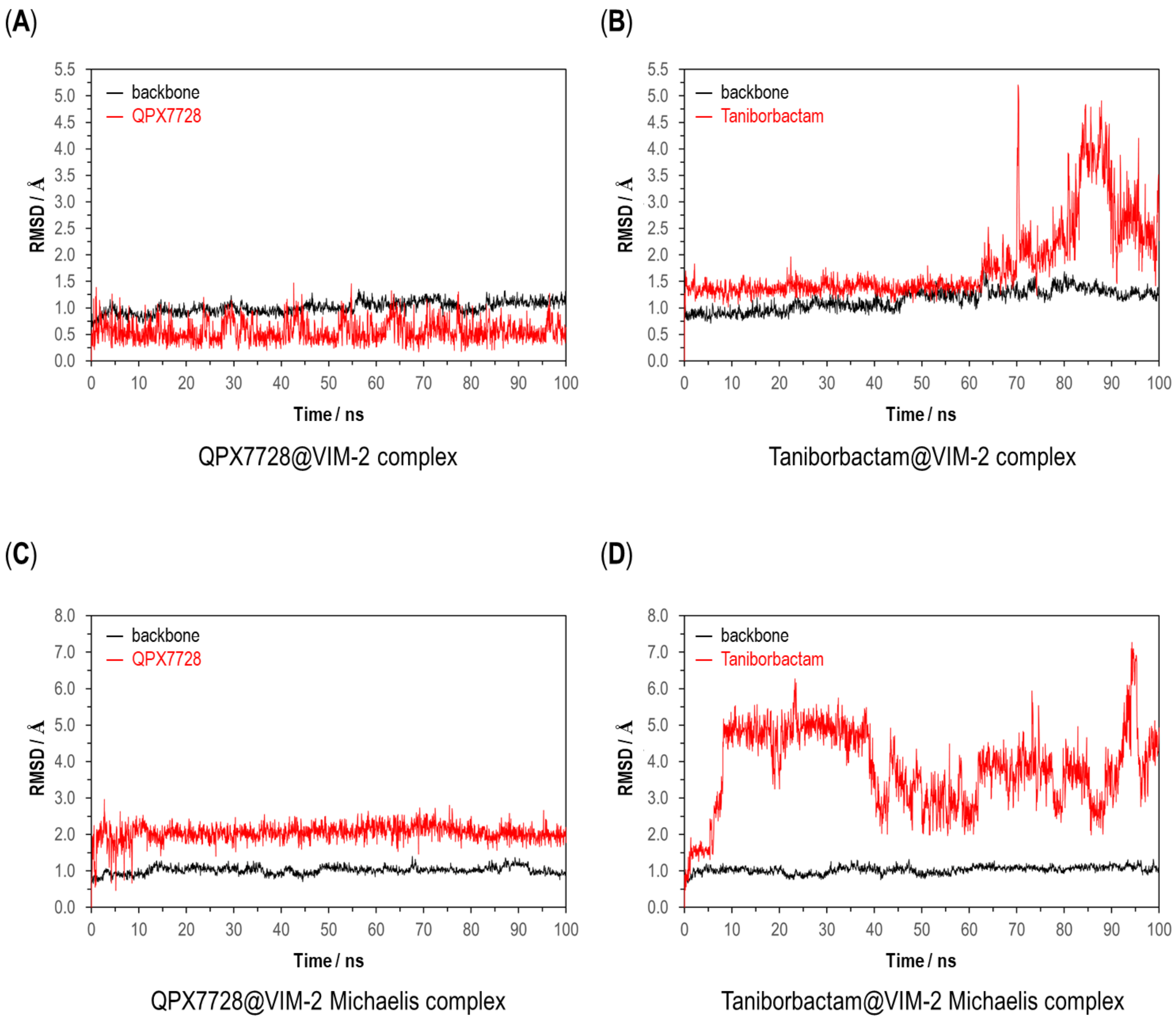

Figure S13. RMSD plots for the protein backbone (Cα, C, O and N atoms, black lines) and the modified ligands (heavy atoms, red lines) calculated from the MD simulations of the complexes: (**A**) QPX7728@VIM-2; (**B**) taniborbactam@VIM-2; (**C**) QPX7728@VIM-2Michaelis complex; (**D**) taniborbactam@VIM-2 Michaelis complex. Note how QPX7728 is remarkably more stable than taniborbactam when binding the VIM-2 active site for Michaelis complex formation.

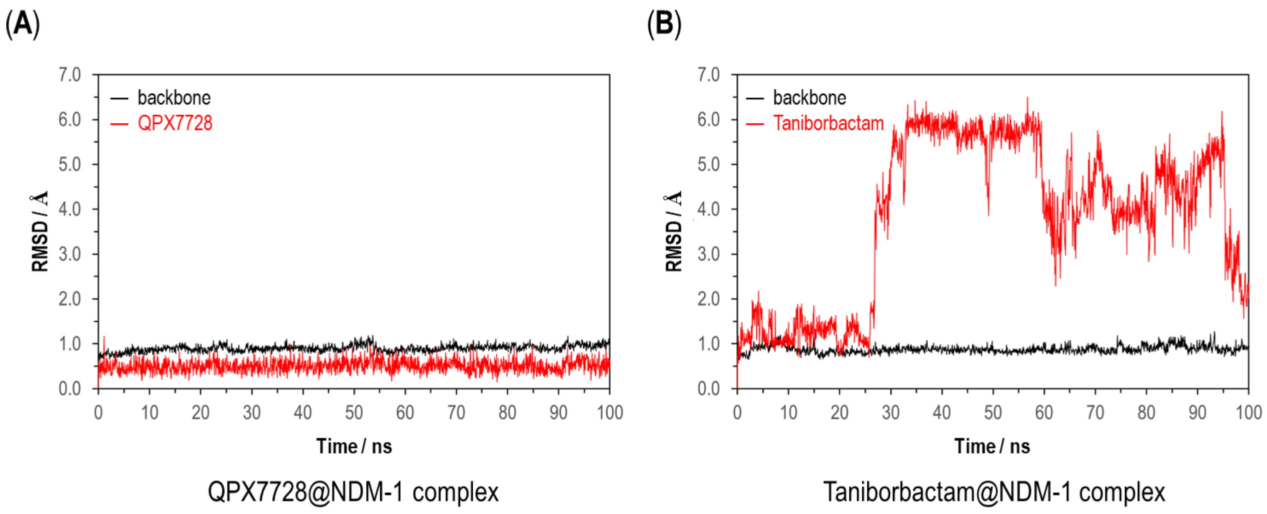

Figure S14. RMSD plots for the protein backbone (Cα, C, O and N atoms, black lines) and the modified ligands (heavy atoms, red lines) calculated from the MD simulations of the complexes: (**A**) QPX7728@NDM-1; (**B**) taniborbactam@NDM-1. The high rmsd values of the modified taniborbactam are due to the large variety of arrangements of its flexible amide substituent, which is absent in QPX7728.
